# Simple, fast, reliable: multiplex digital PCR quantification of 19 genetically modified soybean events

**DOI:** 10.1080/21645698.2026.2635816

**Published:** 2026-02-27

**Authors:** Amadej Jelenčič, Dejan Štebih, Tina Demšar, David Dobnik

**Affiliations:** a Department of Biotechnology and Systems Biology, National Institute of Biology, Ljubljana, Slovenia; b Jozef Stefan International Postgraduate School, Ljubljana, Slovenija

**Keywords:** Genetically modified (GM) crops, transgenic, soybean, Glycine max (L.) merr, quantification, digital PCR (dPCR), multiplex, multi-target

## Abstract

Plant genetic engineering represents an important aspect of modern agriculture, and new genetically modified (GM) crop varieties are entering the market on a regular basis. This necessitates the development of high throughput multi-target analytical methods to detect and quantify their presence for regulatory compliance. In this study, we present a multiplex dPCR method for discriminative quantification of 19 GM soybean events and the lectin (*Le1*) endogene on a nanowell plate-based all-in-one dPCR system. The method consists of four 5-plex assays, taking advantage of the platform’s multiple fluorescence detection channels. The assays complied with the minimum performance requirements in terms of specificity, trueness, precision, sensitivity and dynamic range, making them suitable for use in routine detection and quantification of GM crops. This method represents the most comprehensive multi-target GM soybean quantification approach to date without the need for prior screening and features a simplified workflow, making it suitable for widespread adoption. Our study sets a precedent for rapid and straightforward development of multiplex dPCR GM crop quantification assays to address the evolving demands of regulatory monitoring.

## 1. Introduction

Plant genetic engineering represents an important tool to improve productivity, reduce costs, and lessen the environmental impact of agricultural production.^1,2^ The relatively recently developed approaches of genome editing are starting to be widely utilized in plant breeding,^3,4^ and plant varieties developed in this way are starting to enter the market.^5^ Nevertheless, the so-called genetically modified (GM) crops obtained using the more traditional approach of transgenesis ^6^ are still a major part of agricultural systems, with new GM varieties entering the market on a regular basis.^5,6^

Despite evidence of the safety and utility of GM crops,^1,2^ significant public concern about their usage remains. Legislative frameworks regulating the use and marketing of GM crops have been established in most parts of the world to address concerns about their impact. These regulations ensure traceability, facilitate consumer choice, and protect market segments that prohibit GM crops, such as organic farming.^7,8^

Regulations regarding GM crops differ across the world, with the European Union (EU)’s regulations being among the strictest.^9^ GM plant varieties fall into the category of GM organisms (GMOs) and are only approved for marketing or cultivation after extensive testing of health and environmental safety. Clear labeling is required if they are present above certain minimal thresholds in food and feed products (0.9% mass fraction per ingredient for approved GM events in food and feed and 0.1% mass fraction for events with expired or pending authorization in feed), and marketing of unauthorized GM varieties is forbidden.^8,10^ To comply with regulations, precise, accurate, and robust methods for detection and quantification of GM material are required for characterization of food and feed samples. Currently, methods validated and published by the EU Reference Laboratory for Genetically Modified Food and Feed (EURL GMFF), based on quantitative polymerase chain reaction (qPCR) represent the gold standard in this area,^11^ exhibiting excellent sensitivity and robustness. Nevertheless, their utility in terms of cost- and labor-effectiveness is hindered by qPCR's limited capability for multiplexing (i.e. quantification of multiple targets in the same reaction).^12,13^ Initial screening for presence of genetic elements common in transgenic inserts ^14,15^ reduces the costs and time spent on analysis, but qPCR might still not be the most effective approach in cases of positive screening results.^16,17^

Digital PCR (dPCR) is a method that enables absolute quantification of nucleic acids by partitioning the reaction mixture into thousands of small partitions, eliminating the need for a standard curve.^18^ A multitude of dPCR platforms are commercially available, with the majority accomplishing the partitioning using pre-made partitions on a microfluidic chip/plate ^19,20^ or by generating water-in-oil emulsion droplets.^13,18^ While the costs of performing simplex dPCR exceed those of qPCR, multiplexing allows it to become more cost efficient.^21^ Furthermore, the superior analytical performance and time-efficiency of dPCR make it an outstanding choice for the quantification of GM events in food and feed.^13,16,17,22^

dPCR possesses several advantages compared to qPCR, as quantification is not dependent on certified reference materials (CRMs), is less affected by PCR inhibition and enables easier implementation of multiplexing.^13,16,17^ The latter advantage is especially important, as the number of GM events keeps growing, making essential the simultaneous detection and quantification of numerous targets. dPCR-based methods might provide a time- and cost-effective solution to that challenge. While several qualitative multiplex assays have been developed for qPCR,^14,23,24^ the development of quantitative methods has proven difficult, especially with higher levels of multiplexing.^16^ The complexity introduced by reliance on CRM-based standard curves, differing amplification efficiencies between samples, sensitivity to PCR inhibitors, and competition for reagents makes the development and validation of quantitative multiplex qPCR methods exceedingly difficult.^25^ Conversely, dPCR does not possess those limitations. Direct quantification obviates the need for standard curves. Furthermore, dPCR analysis is based on end-point signal readout (as opposed to real-time readouts of qPCR) which greatly reduces the impact of amplification efficiency and PCR inhibitors. Competition for reagents is greatly reduced by compartmentalization of the reaction mixture.^18,25^ High sensitivity, specificity, precision and the ability of simultaneous detection of multiple fluorescent dyes are additional characteristics that make dPCR an optimal choice for quantitative multiplexing.^13,16,17,21,22^

The use of dPCR for multiplex quantification of GM lines has been demonstrated in maize (*Zea mays* L.) ^17,26^ and soybean (*Glycine max* (L.) Merr.).^16,22^ These approaches allowed for the determination of the quantity of GM material in the sample but did not discriminate between individual GM events. This is sufficient for the purpose of analysis in accordance with the EU labeling regulation, which requires quantification of all authorized events per plant species.^27^ Nevertheless, high-throughput quantification of individual GM events in a sample might be desirable for some applications and some regulatory bodies prefer this kind of information, e.g., to provide better traceability information, and allow for more accurate labeling. Multiplex dPCR has been used to discriminately quantify GM events in maize,^13^ soybean and canola (*Brassica napus*).^28^ As the dPCR systems used in those studies only allowed for simultaneous detection of two fluorescent dyes, multiplexing was achieved by varying the concentrations of primers and probes in the so-called amplitude multiplexing approach.^29^ While this has proven viable, there is a possibility of false positive results. Recently, dPCR systems featuring up to seven optical channels for simultaneous detection of multiple fluorescent dyes have become available.^30–37^ This enables relatively simple multiplexing as each target can be quantified in a separate channel. Bogožalec Košir *et al*. have formulated and tested a 6-plex and two 4-plex assays for discriminative GM soybean detection and quantification, proving the feasibility of such an approach.^22^

The multiplex dPCR methods reported to date feature either non-discriminative multiplex quantification or discriminative quantification of a limited number of targets. Furthermore, the dPCR systems used in previous studies perform reaction compartmentalization, temperature cycling and fluorescence readout steps in separate instruments, requiring additional sample handling between those steps. Moreover, some steps require specialized pipetting technique, increasing the complexity of the procedure and operator training investment. The present study aimed to significantly streamline and speed up the experimental procedure while also expanding the number of discriminately quantified GM targets. Using an all-in-one dPCR system, we developed and tested a method that quantified all 17 GM soybean events authorized in the EU at the time of method development, one event with pending approval (DBN-09004–6) and one event with expired authorization (356043).^38^ We analyzed the samples by four 5-plex assays, one of them quantifying four GM soybean events and the soybean endogene *Le1*, which served as the reference gene,^16^ and the other three quantifying five events each. We characterized the assays in terms of their performance characteristics which showed that they are suitable for their intended purpose.

## 2. Materials and Methods

### 2.1. Test Material

For characterization of the assays, dilutions and mixtures of DNA extracted from GM soybean CRMs were prepared. For specificity assessment, a mixture of non-soybean GM event CRMs was prepared. All CRMs were obtained either from American Oil Chemists’ Society (AOCS) or European Commission, Joint Research Center, Directorate F – Health, and Food (EC JRC Directorate F). Soybean CRMs obtained from AOCS included AOCS 0112-A2 (100% [1000 g/kg] SYHT0H2), AOCS 0215-A (100% [1000 g/kg] MON87751), AOCS 0911-D (100% [1000 g/kg] BPS-CV127-9), AOCS 0809-B2 (100% [1000 g/kg] MON87769), AOCS 0809-A2 (99.2% [992 g/kg] MON87701), AOCS 0311-A2 (99.9% [999 g/kg] MON87708), AOCS 0707-C8, and AOCS-0707-C9 (100% [>999.99 g/kg] A5547-127), AOCS 0707-B14 (100% [>999.99 g/kg] A2704-12), AOCS 0906-B2 (100% [1000 g/kg] MON89788), AOCS 0210-A2 (100% [1000 g/kg] MON87705), AOCS 0610-A6 (100% [>999.99 g/kg] FG72), and AOCS 0911-A (non-modified soybean). Soybean CRMs obtained from EC JRC Directorate F included ERM-BF441b (100% [1000 g/kg] DBN-09004–6), ERM-BF443b (100% [1000 g/kg] GMB151), ERM-BF426d (10.0% [100 g/kg] 305423), ERM-BF410bp (>98.5% [>985 g/kg] 40–3-2), ERM-BF432d (10.0% [100 g/kg] DAS-68416–4), ERM-BF425d (10.0% [100 g/kg] 356043), ERM-BF437b (>98.6% [>986 g/kg] DAS-81419–2), and ERM-BF436b (>98.6% [>986 g/kg] DAS-44406–6). Non-soybean CRMs obtained from AOCS included AOCS 0304-B (pure homozygous GT73 canola), AOCS 1011-A (pure homozygous MON88302 canola), AOCS 0711-D3 (pure homozygous Topas 19/2 canola), AOCS 1116-A (100% [>999.99 g/kg] Ms11 canola), AOCS 0306-F8 (100% [>999.99 g/kg] Ms8 canola). AOCS 0306-G7 (100% [>999.99 g/kg] Rf3 canola), AOCS 0117-A (non-modified canola), AOCS 0306-I8 (pure homozygous LLRice62 rice), AOCS 0406-D (pure heterozygous MON 88,017 maize), AOCS 0906-E (pure heterozygous MON89034 maize), AOCS 1208-A (pure heterozygous MIR162 maize), AOCS 0709-A (pure heterozygous MON87460 maize), AOCS 0407-B (100% [1000 g/kg] GA21 maize), AOCS 030-H5 (pure homozygous T25 maize), AOCS 0512-A (pure heterozygous MON87427 maize), AOCS 0216-A (99.7% [997 g/kg] MON87403 maize), AOCS 1114-C (98.0% [980 g/kg] MZHG0JG maize), and AOCS 0818-A2 (99.9% [999 g/kg] MON87419 maize). Non.soybean CRMs obtained from EC JRC Directorate F included ERM-BF422b (>97,9% [>979 g/kg] 281–24-236 ×3006–210-23 cotton), ERM-BF419b (100% [1000 g/kg] H7-1 sugar beet), ERM-BF421b (100% [1000 g/kg] EH92-5271 potato), (>98.8% [>988 g/kg] 73,496 rapeseed), ERM-BF411f (5.0% [50 g/kg] Bt176 maize), ERM-BF418d (9.86% [98.6 g/kg] DAS1507 maize), ERM-BF433d (10.0% [100 g/kg] DAS-40278–9 maize), ERM-BF427d (10.0% [100 g/kg] DP-98140–6 maize), ERM-BF423d (9.85% [98.5 g/kg] MIR604 maize), ERM-BF420c (9.8% [98 g/kg[3272 maize), ERM-BF413gk (9.9% [99 g/kg] MON810 maize), ERM-BF416d (9.85% [98.5 g/kg] MON863 maize), ERM-BF424d (9.87% [98.7 g/kg] DAS59122 maize), ERM-BF412f (4.89% [48.9 g/kg] Bt11 maize), ERM-BF415f (4.91% [49.1 g/kg] NK603 maize), ERM-BF438b (>98.6% [>986 g/kg] VCO-01981–5 maize), and ERM-BF439b (>98.6% [>986 g/kg] 4114 maize). Non-modified papaya DNA was extracted from dried fruit. A proficiency test sample (sample A) and routine diagnostics soybean samples (samples B-D) were used for the determination of fitness for purpose.

### 2.2. DNA Extraction

The DNA of all CRMs and samples A and B were extracted by the cetyltrimethylammonium bromide (CTAB) method, with RNase-A and proteinase-K for removal of RNA and protein, respectively, as described in Annex A.3 of ISO21571:2005. The DNA of samples C and D were extracted by the NucleoSpin®Food kit (Macherey-Nagel GmbH & Co. KG, Düren, Germany), according to manufacturer instructions. 1 g of starting material was used for CRMs obtained from AOCS, and 0.5 g for CRMs obtained from EC JRC Directorate F. Samples extracted with CTAB were eluted in 150 or 200 μL of water, according to sample type, based on in-house experience. Samples extracted with NucleoSpin were eluted in 200 μL of water. After elution, 500 μL of water was added to sample C and 800 μL to sample D, based on the sample type. All extractions included a negative extraction control where water was used instead of sample. Dilutions of the extracted stock DNA solutions were prepared with 2 µg/mL sheared calf thymus DNA (Cytiva, Marlborough, MA, USA) in nuclease- and protease-free water (Sigma-Aldrich Chemie GmbH, Taufkirchen, Germany). All samples were stored below −15°C until further use.

### 2.3. In silico Specificity and Primer/Probe Interaction Prediction

Three 5-plex assay combinations were designed based on the previous combinations of individual assays in multiplex format, for which specificity had already been assessed.^16,22^ The fourth assay comprised a novel combination of primers and probes. *In silico* analyses were performed for all multiplex assays to determine specificity and potential interactions between primers and probes. For the latter, the software Autodimer was used with default settings.^39^ For *in silico* specificity analysis, the software MFEPrimer was used ^40^ with default settings. The primers used in each 5-plex assay were tested against the genomes of soybean, maize, oilseed rape, rice, and wheat. A potential amplicon was considered problematic if both primers possessed melting temperatures (Tm) over 50°C.^41^

### 2.4. Digital PCR on the QIAcuity Platform

Digital PCR measurements on QIAcuity One instrument (QIAGEN, Hilden, Germany) were performed on 8,5k 24- and 96-well QIAcuity nanoplates, with QIAcuity Probe PCR Kit (QIAGEN, Hilden, Germany). The 12 μL reaction mixtures consisted of Probe PCR master mix, primer-probe mix, nuclease-free water, and 4 μL DNA sample. The final concentrations of primers and probes were the same as specified by reference qPCR methods for each event (Table S1).^11^ The reporter dyes and quenchers were selected to enable multiplex detection in separate optical channels and were in some cases different than in the reference qPCR methods. Double-quenched probes were used when possible, to reduce background fluorescence. The PCR cycling conditions consisted of 2 min at 95°C followed by 40 cycles of 15 s at 95°C and 30 s at 60°C. The imaging exposure duration and gain settings for all channels were left on default. QIAcuity Software Suite, version 2.5.0.1 (QIAGEN, Hilden, Germany) was used for result analysis. Further calculations were performed in Excel (Microsoft Corporation, Redmond, WA, USA). Fluorescence thresholds were set manually in a 1D amplification plot view with non-template controls (NTCs) as a guide in all channels for simplex assays and in green, yellow, and orange channels for 5-plex assays (Figures S1-S4). Fluorescence thresholds in red and crimson channels for 5-plex assays were set in 2D view to account for fluorescence bleed-through from red to crimson channel (see Figure S1). Unless otherwise specified, the results are presented as the mean target copy number per analyzed reaction volume. These were calculated by multiplying the concentration displayed by the software with the analyzed volume per well. Copy number ratios were calculated by dividing mean target GM event copies per reaction with mean reference gene *Le1* copies per reaction for a given sample. GM percentages (GM%) are given as mass fractions (m/m) and are obtained by applying the relevant conversion factors (CF) to the copy number ratios, according to CF for certified references materials guidance document, version 13.^42,43^ Where applicable, coefficients of variation (CV) were calculated by dividing the standard deviation of replicates by their average. Biases were calculated by subtracting the reference value from the tested value and dividing the result by the reference value.

### 2.5. Specificity Assessment

For in vitro specificity assessment, mixtures containing approximately equal amounts of events detected by each 5-plex were prepared. Each mixture was tested by assays that do not detect the events it contains. Furthermore, a DNA mixture of canola events T45, RF3, MON88302, MS8, GT73, and 73,496, cotton event 281x3006, sugar beet event H7-1, rice event LL62, potato event EH92, various GM maize events, and non-GM canola and papaya was prepared and tested with the four 5-plex assays. Each reaction was performed in triplicate.

### 2.6. Comparison of Simplex and Multiplex Assays

For comparison of results between simplex and multiplex assays, and initial characterization of the materials, quantification of the 19 events and endogene *Le1* was performed in simplex and multiplex dPCR reactions on the QIAcuity platform. CRM stock DNA for individual events was diluted to a working concentration deemed suitable for quantification based on previous experience. For most events, two dilutions (20x and 80x) of the DNA working solution were tested with event-specific and *Le1*-specific simplex assays and the corresponding 5-plex assay. The CRMs for events 305423, DAS-68416–4, and 356043 each contained 10% of the corresponding event. Therefore, different dilutions had to be used to fall within the predicted dynamic range for target event and *Le1* quantification in each material. For these targets, 2x, and 4x dilutions of the DNA working solution were used to quantify the events, and 32x and 128x dilutions to quantify *Le1*. Additionally, four DNA mixtures were prepared, each containing the GM events targeted by a given 5-plex assay (see Table S2). Events targeted by 5-plexes 2–4 were also tested with 5-plex 1 for *Le1* quantification. Each reaction was performed in duplicate. Results were normalized to undiluted sample conditions by applying appropriate dilution factors.

### 2.7. Determination of Dynamic Range and Linearity

For the determination of dynamic range, a mixture of DNA was prepared, containing approximately 2600–3200 target copies of each GM soybean event per reaction (Mix D1), along with dilutions of this mixture (Mixes D2-7), with the last dilution containing <1 copy per reaction of each event. These dilutions were tested in 16 technical replicates (4 replicates on 4 separate days). The results from this dilution series were used for evaluation of linearity.

### 2.8. Determination of Limits of Detection and Quantification

Limits of quantification (LOQ) for target GM soybean events and limit of detection (LOD) for *Le1* under symmetric conditions were determined using the results of the dynamic range experiment (see Determination of Dynamic Range and Linearity). LOQ for *Le1* was determined by testing three dilutions of a mixture of events DBN-09004–6, GMB151, SYHT0H2, and MON87751. A single dilution (250x) of Mix D1 (see Determination of Dynamic Range and Linearity) was tested with each assay in 16 technical replicates to determine the symmetric LOD for target GM soybean events. Additionally, LOD and LOQ for the GM soybean events were determined under asymmetric conditions on samples with low target copy numbers of the tested events and high copy numbers of the other targets. For each 5-plex assay, 10 DNA mixtures of events quantified by that assay were prepared. Each mixture contained low copy numbers (~4–80 copies per reaction) of 2 or 3 targets and high copy numbers of the other targets (~9500–25000 total copies of background targets per reaction), arranged in a way to allow for determination of asymmetric LOD and LOQ. Each DNA mixture was tested in 9 technical replicates with the corresponding assay.

### 2.9. Determination of Robustness

We tested the robustness of the method by applying small deliberate alterations to two parameters, annealing/extension temperature and concentration of primers and probes. For the former, we tested temperatures of 59°C and 61°C in addition to the usual 60°C. For the latter, we decreased and increased the concentration of primers and probes in the reaction by 10%. For each condition, two dilutions of a DNA mixture of all tested soybean events were tested, containing approximately 80 and 30 copies of each target event, respectively. Each dilution was tested in two technical replicates.

### 2.10. Determination of Fitness for Purpose

A proficiency test sample (sample A), and three routine diagnostic samples (samples B-D) were analyzed by the four 5-plexes. The experimental setup varied, based on sample availability. For sample A, a 10x dilution of a single DNA extract was tested in 4 replicates. For samples B and C, 2 dilutions (5x and 10x, and 2x and 6x, respectively) of 2 DNA extracts were tested, each in 2 technical replicates (8 reaction wells in total). For sample D, a 10x dilution of a single DNA extract was tested in 3 technical replicates.

## 3. Results

### 3.1. Assay Design

EU reference methods for event-specific GM soybean quantification^11^ were combined into four 5-plex assays (Table S2). The four most recently developed reference assays (for events DBN-09004–6, GMB151, SYHT0H2, and MON87751) and the reference assay for *Le1* were combined to obtain the first 5-plex assay (5-plex 1). Multiplex assays developed by Bogožalec Košir et al. (2017, 2023) were used as a guide in designing the other three assays (5-plexes 2–4), not to introduce too many new combinations, as specificity for some was already evaluated in the previous study. The targets detected by each 5-plex assay were as follows (see Table S2): 5-plex 1 – DBN-09004–6, GMB151, SYHT0H2, MON87751, and *Le1*; 5-plex 2 – 305423, BPS-CV127-9, MON87769, MON87701, and MON87708; 5-plex 3 - 40–3-2, A5547-127, A2704-12, MON89788, and MON87705; 5-plex 4 – FG72, DAS-68416–4, 356043, DAS-81419–2, and DAS-44406–6.

### 3.2. Specificity

**Figure 1.**
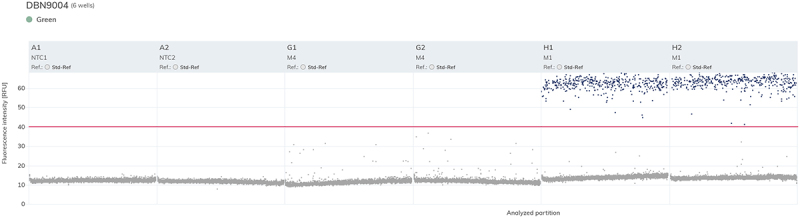
Low-level fluorescence in some partitions for DBN-09004–6 target when using 5-plex 1 assay and complex mixtures of soybean GM lines. Wells A1 and A2 contained the non-template controls (NTC), wells G1 and G2 contained a DNA mixture of GM soybean events FG72, DAS-68416–4, 356043, DAS-81419–2 and DAS-44406–4, and wells H1 and H2 contained a DNA mixture of GM soybean events detected by 5-plex 1 (DBN-09004–6, GMB151, SYHT0H2 and MON87701). Individual partitions are plotted along the x-axis, and the y-axis represents the fluorescence intensity in relative fluorescence units (RFU). The threshold (red line) is set in a way to separate true positives from negative signal.

*In silico* specificity assessment against the genomes of soybean, maize, oilseed rape, rice, and wheat was performed using the software MFEprimer.^40^ We found that no significant nonspecific amplification is expected. Dimer formation prediction using the software Autodimer^39^ found that no dimers between primers and probes are expected to form in the 5-plexes. Specificity of each 5-plex assay was also tested experimentally by analyzing mixtures of non-target soybean events in addition to a mixture of non-soybean GM events. No nonspecific amplification was observed for 5-plexes 2–4. Assay 5-plex 1, when testing the mixture of events FG72, DAS-68416–4, 356043, DAS-81419–2 and DAS-44406–4 (targeted by 5-plex 4), produced a number of partitions in the green channel (for event DBN-09004–6) with fluorescence higher than that of the negative population of partitions but lower than that of true positives (Figure 1). It was determined that the presence of events DAS-68416–4 and 356043 was the main cause of the appearance of intermediate fluorescence partitions (Figure S5). As the assay offers good resolution between true positive and negative populations, it was decided that fluorescence threshold can be set above the fluorescence of the intermediate partitions without losing accuracy of quantification (see Figure 1). We therefore concluded that the method possesses sufficient specificity for its intended purpose.

### 3.3. Comparison Between Simplex and Multiplex Assays

Quantification by simplex and multiplex assays was compared by testing solutions containing single GM events. All four 5-plex assays showed results comparable with simplex assays, with bias under 25% for all events tested (Table 1; Tables S3 and S4). The 5-plex assays were therefore deemed suitable for further experiments.

**Table 1.** Comparison between 5-plex and simplex dPCR assays.

GM event	Bias 5-plex/simplex single events (%)^a,b^	Bias 5-plex/simplex mixture (%)^a,c^
DBN-09004–6	−4.0	0.3
GMB151	2.5	12.0
SYHT0H2	−0.22	−4.2
MON87751	−5.7	0.1
Le1	5.6	6.6
305423	4.0	−2.0
BPS-CV127-9	−1.3	4.7
MON87769	−4.0	7.5
MON87701	−0.1	−5.5
MON87708	4.0	−18.4
40–3-2	8.5	10.0
A5547-127	1.9	11.8
A2704-12	−9.9	8.6
MON89788	5.4	14.4
MON87705	3.9	−8.2
FG72	10.3	−5.3
DAS-68416–4	6.1	7.8
356043	−10.9	13.5
DAS-81419–2	−12.7	8.3
DAS-44406–6	−14.2	6.8

^a^Values were obtained by calculating the bias in terms of target copy numbers per reaction between 5-plex dPCR assays and the relevant simplex assays.

^b^Bias for Le1 was <25% for all tested samples (dilutions of individual GM soybean event certified reference material (CRM) DNA). Here, results for sample containing DNA of event DBN-09004–6 are shown.

^c^Bias for Le1 was <25% for all tested samples (Soybean DNA mixtures 1–4 corresponding to GM events quantified 5-plexes 1–4). Here, results for DNA mix 1 are shown, containing DNA of events DBN-09004–6, GMB151, SYHT0H2, and MON87751.

### 3.4. Trueness

To determine the method’s trueness, the multiplex dPCR experimental data from simplex-multiplex comparison were used to calculate GM percentages for each CRM and compared with the CRMs’ assigned percentages. The bias between multiplex dPCR results and assigned values was under 25% for all events tested (Table S5), which is in accordance with the GMO analytical method performance guidelines by the European network of GMO reference laboratories (ENGL).^44^

### 3.5. Dynamic Range and Linearity

The dynamic range of the method was determined by testing DNA mixes D1-7, containing between 0 and 3300 target copies per reaction for each GM event. The dynamic range of all targeted GM events spanned at least between 40 and 2520 copies (Table 2, Tables S6-S9), complying with the requirements set by ENGL guidelines.^44^ The tested dilutions were not suitable for determining the lower bound of dynamic range for *Le1*. The latter was determined in a separate reaction run (see subsection 3.6) to be 39 cp/rxn (the LOQ value obtained for *Le1*). The dilutions containing more than 56,000 copies of *Le1* per reaction (ENGL-defined upper limit for endogene) were not accurately quantified due to the limitations of the plate setup used (~8200 analyzed partitions per reaction). This can easily be mitigated by diluting the tested sample and therefore does not present a significant barrier to accurate GM% quantification. The linearity of all assays was found to be sufficient across the dynamic range with R^2^ > 0.99 for all events tested (Figures S6-S9).

**Table 2.** Upper and lower bounds of the quantitative dynamic range (DR) in copies per reaction (cp/rxn) for the 5-plex assays. The symmetric limits of quantification (LOQ) are equal to the lower bound of DR.

GM event	DR upper bound (cp/rxn)	DR lower bound/LOQ (cp/rxn)
DBN-09004–6	2576	28
GMB151	2923	36
SYHT0H2	2582	34
MON87751	2779	35
*Le1*	16,881	39
305423	2817	27
BPS-CV127-9	2756	33
MON87769	2600	28
MON87701	2946	31
MON87708	2777	31
40–3-2	3249	36
A5547-127	3003	34
A2704-12	2956	31
MON89788	2764	35
MON87705	2797	27
FG72	2839	36
DAS-68416–4	2734	30
356043	2640	29
DAS-81419–2	2704	28
DAS-44406–6	2795	31

### 3.6. Sensitivity and Precision

First, the LOQ and LOD for all targets were determined under symmetric conditions with approximately equal concentrations of each target. The LOQs for the GM soybean events were determined using data from the dynamic range experiment and are equal to the lower bound of the dynamic range for each event (27–36 cp/rxn; see Dynamic Range and Linearity, Table 2, Tables S6-S9). The LOD for *Le1* was obtained from the same data and was found to be 8 cp/rxn (see Table 2, Table S6). The LOQ for *Le1* was obtained by testing dilutions of a mixture of 4 GM soybean events (DBN-09004–6, GMB151, SYHT0H2, and MON87751) and was determined to be 39 cp/rxn (see Table 3, Table S11). The LODs for GM soybean events were determined using a 250x dilution of mix D1 (see Dynamic Range and Linearity). The results showed no negative replicates for any event, so the obtained values (13–19 cp/rxn) were determined to be the LOD for each target event (Table S10). Additionally, LOD and LOQ for GM soybean event targets were determined for multiplex assays under asymmetric conditions by testing solutions containing decreasing target copy numbers of the quantified event in a high copy number (9500–25000) background of other targets. LOD and LOQ for *Le1* were not determined under asymmetric conditions as that would not be relevant to real-world samples. All events displayed asymmetric LOQ under 50 copies per reaction (Table 3, Tables S11-S14). The asymmetric LOD for the individual GM events was determined to be between 5–16 copies per reaction (see Tables S11-S14). All LOD values are below the ENGL-defined limit of 25 copies per reaction.^44^

**Table 3.** Asymmetric limits of detection (LOD) and quantification (LOQ) in copies per reaction (cp/rxn) for the 5-plex assays.

GM event	Asymmetric LOQ (cp/rxn)	Asymmetric LOD (cp/rxn)
DBN-09004–6	40	11
GMB151	34	7
SYHT0H2	45	5
MON87751	16	16
Le1	39^a^	8^a^
305423	16	16
BPS-CV127-9	20	9
MON87769	36	9
MON87701	42	9
MON87708	18	8
40–3-2	44	5
A5547-127	23	6
A2704-12	39	5
MON89788	27	5
MON87705	25	10
FG72	29	15
DAS-68416–4	21	10
356043	39	8
DAS-81419–2	33	6
DAS-44406–6	18	9

^a^LOD and LOQ for *Le1* were only determined in symmetric conditions.

### 3.7. Robustness

To test the method’s robustness, the assays were tested under deliberate modifications to the reaction conditions. We tested modifications to the primer annealing temperature (±1°C) and concentration of primers and probes (±10%). The CV across all tested conditions did not exceed 25% for any target tested (Table S15). The bias exceeded 30% in 1 instance for the event SYHT0H2 when the temperature was decreased by 1°C. This, however, could be due to the relatively low number of technical replicates tested and would presumably improve with a higher number of replicates. The average bias for this individual target at all tested conditions was 18.5%. Consequently, the results do not seem to be caused by a systemic bias associated with the particular assay. Overall, we found the method to possess sufficient robustness.

### 3.8. Fitness for Purpose

To evaluate the method’s fitness for purpose, a proficiency test sample (sample A) was tested and the results compared with the assigned values from the proficiency test. Additionally, three routine diagnostic samples (samples B-D) were tested and the results compared with previous results from our laboratory.

In sample A, multiplex dPCR detected 1.11% of event BPS-CV127-9, compared to an assigned value of 1.18% from the proficiency test, resulting in a bias of −5.9%.

For sample B, the results were compared with the qPCR-based analyses performed during the original diagnostic characterization of the sample. 5-plex dPCR detected the same events as qPCR. For the quantified events (A2704-12, 305,423 and 40–3-2), the bias of dPCR compared to qPCR was under 10% for all three events, falling well under the ENGL-defined limit of 25% (Table 4). Multiplex dPCR confirmed the presence of the event MON89788 below 0.1% that had also been detected but not quantified by qPCR.

Sample C had previously been assessed qualitatively by qPCR and quantitatively by simplex dPCR assays on the same platform that we used in our experiments. All 3 methods detected the same events and the quantification bias between 5-plex and simplex dPCR assays for most events is below 25% (Table 5). The bias for event DAS-44406–6 is just slightly over that, at −25.4% bias of 5-plex to simplex. It should be noted that the detected copies per reaction for this event (4–16) were lower than the assay’s LOQ, explaining the higher variability.

**Table 4.** Result comparison between 5-plex dPCR and qPCR for diagnostic sample B.

GM event^a^	GM% qPCR	GM% dPCR 5-plex	Bias dPCR/qPCR (%)
305423	0.28	0.26	−6.0
40–3-2	1.37	1.35	−1.6
A2704-12	0.42	0.45	6.5
MON89788	Below 0.1	Below 0.1	NA

^a^GM events that are not shown were negative with both methods. NA – not applicable.

For most events in sample D, only qualitative detection had been performed by qPCR, so the results could only be compared qualitatively. All the events detected by qPCR were also confirmed by 5-plex dPCR and the others were undetected by both methods. Additionally, quantification by qPCR was performed for the event MON87701, and the results agreed with 5-plex dPCR (Table 6).

**Table 5.** Result comparison between 5-plex and simplex dPCR for diagnostic sample C.

GM event^a^	GM% dPCR simplex	GM% dPCR 5-plex	Bias 5-plex/simplex (%)
MON87751	3.07	3.44	12.11
MON87701	80.3	77.64	−3.29
MON87708	3.83	3.35	−12.59
40–3-2	12.96	11.98	−7.62
MON89788	54.45	53.88	−1.04
DAS-81419–2	0.47	0.36	−23.01
DAS-44406–6	0.50	0.37	−25.35

^a^GM events that are not shown were negative with both methods.

**Table 6.** Result comparison between 5-plex dPCR and qPCR for diagnostic sample D.

GM event	Result qPCR	GM% dPCR 5-plex	Bias dPCR/qPCR (%)
MON87751	Positive	6.7	NA
MON87701	70.4	74.4	5.7
MON87708	Positive	6.0	NA
40–3-2	Positive	10.4	NA
MON89788	Positive	52.6	NA
DAS-81419–2	Positive	0.9	NA
DAS-44406–6	Positive	0.8	NA

GM events that are not shown were negative with both methods. NA – not applicable.

## 4. Discussion

In this study, we present a highly time- and cost-efficient multiplex dPCR-based method for simultaneous quantification of 19 soybean GM events and the endogene, *Le1*. This represents a significant upgrade on the work of Bogožalec Košir *et al*., who established methods for non-discriminative and discriminative multiplex quantification of GM soybean using dPCR.^16,22^ The method described herein expands this capability by allowing for discriminative quantification of a significantly larger number of GM events. By quantifying all EU authorized GM soybean events at the time of development, an expired event, and an event with pending approval, the assays comprehensively cover the scope of GM soybean varieties likely to be present in the EU. Demeke & Eng developed a multiplex dPCR method for detection of the same 19 events using a combination of element-specific and event-specific assays.^45^ Although the method is useful for detecting these events, discriminative quantification is only possible for the 4 events in the event-specific multiplex assay.

Our approach represents a significant step forward by providing discriminative quantification of a large number of targets. Furthermore, we utilized a different dPCR platform than those used in the aforementioned studies, providing further proof that quantitative multiplexing can become time and cost-effective. A nanowell plate-based all-in-one dPCR system with five fluorescent detection channels was used, where sample compartmentalization, temperature cycling, and fluorescence detection are all performed automatically on the same machine, greatly simplifying the workflow and minimizing hands-on time. The experimental protocol requires no specialized sample handling, allowing it to be performed by operators familiar with qPCR workflow without additional training.

Additionally, this sets precedent and proves the feasibility of quantification of all GM events of a given species in a small number of reactions using multiplex dPCR. In the future, this could expand to other crop species, and eventually all available GM events will be able to be quantified in a much lower number of reactions compared to the current state. This would both increase the possible analytical complexity and reduce the cost of analyses that can be performed on routine GM diagnostics samples, improving traceability and regulatory compliance of food and feed, as well as potentially providing more information to consumers and producers. Furthermore, our approach makes the initial screening and identification step obsolete, unless unauthorized GMOs must be detected in a sample without any other GMOs present.

When developing multiplex assays, specificity is one of the most important concerns and rigorous testing should be performed to ensure that a given assay really quantifies the correct targets and does not produce a positive signal with non-target DNA sequences.^13,16,17,22^ We tested specificity against the most common agricultural crops *in silico* using the MFEprimer software, which detected no concerning potential amplicons. However, *in silico* analysis is limited by the unavailability of the DNA sequences for some GM events. Due to this fact, we were unable to test the 5-plexes’ specificity against all sequences of GM soybean and other GM crops. Thus, we focused on real-life (*in vitro*) testing scenarios. Out of the total of 20 individual assays/targets, we observed some partitions with low to intermediate fluorescence during *in vitro* specificity testing only for one of the individual targets, when all the soybean GM event targets were present in the sample. As the presence of those partitions can be mitigated by setting the fluorescence threshold above the nonspecific partitions, there was no interference with accurate and specific quantification.

A new qPCR-based reference method became available for quantification of GM soybean event MON-94313–8 with pending EU authorization when this manuscript was being drafted.^46,47^ This both exemplifies the rapid rate at which new GM varieties are entering the market and suggests ways to expand the method presented herein. The new event could easily be quantified by including a simplex dPCR adaptation of the reference method alongside the four 5-plexes. The simplex could be expanded with additional GM event targets as new reference methods become available. Furthermore, a software update for the QIAcuity Software Suite has become available that enables fluorescence detection in six optical channels and two channel combinations, allowing to go up to 8-plex.^48^ Consequently, the method could also be expanded by adding the new target and other potential targets in the future to the existing 5-plex assays.

An important consideration with dPCR assays is the issue of the so-called dead volume, the fraction of the reaction that is not analyzed. While qPCR comprises the analysis of the whole reaction mixture, in dPCR, only a portion is compartmentalized and analyzed.^49,50^ This, in addition to being a source of subsampling variability, also creates ambiguity in the definition of terms used in the context of method performance evaluation. For example, ENGL guidelines ^44^ require digital PCR GMO quantification methods to possess an LOQ under 50 copies per reaction. However, because the whole reaction volume is not analyzed, it is unclear whether the full reaction volume, or only the analyzed portion should be used in this context. The choice of which definition to use can have a large impact on method performance assessment.^51^ In this study, we have chosen to evaluate the method based on the number of target copies per analyzed volume. In this way, the method’s quantification ability in the analyzed volume is comparable to that of other platforms and setups. Quantification with our method would only be compromised in the case when a sample possesses exceedingly low target copy concentrations. However, any other method would struggle with accurate quantification at such low concentration. If such sensitivity were required, DNA extraction could be repeated with a larger amount of starting material or a more efficient extraction method to obtain a sample with a higher concentration.^22^ Furthermore, larger volumes of sample DNA could be added to the reaction to get a larger effective concentration. In our experiments, we added 4 µL of DNA, but theoretically, up to 8.5 µL could be added if using highly concentrated primer-probe mixes. This would have no impact on the method’s LOD or LOQ if copy numbers per total reaction volume were considered but would effectively allow detection and quantification in samples with lower target concentrations. Additionally, QIAcuity 26k Nanoplates could be utilized which possess lower dead volume and allow for greater sensitivity, albeit with lower sample throughput.^49^ If necessary, the method could be adapted to other dPCR platforms, although the fluorescent dyes on some probes would possibly need to be changed to accommodate for differences in optical detection channels. In that case, some additional testing or verification would be necessary to ensure proper performance.

In summary, our study provides an example of streamlined development of high-throughput multiplex dPCR GM crop quantification assays by establishing a method for quantifying 19 GM soybean events likely to be present in the EU. This represents an opportunity for diagnostic laboratories to drastically reduce the number of reactions needed for quantification of all soybean events likely to be present in a given sample. If needed, the method can easily be adopted to other platforms and setups, increasing its versatility and usefulness. Development of similar methods for other GM crops would allow for extensive quantitative characterization of GM food and feed samples in relatively few reactions.

## Supplementary Material

Supplementary information 1_Tables and figures clean.docx

Supplementary information 2_dMIQE2020 checklist.docx

## References

[1] Anderson JA, Gipmans M, Hurst S, Layton R, Nehra N, Pickett J, Shah DM, Souza TLPO, Tripathi L. Emerging agricultural biotechnologies for sustainable agriculture and food security. J Agric Food Chem. 2016;64(2):383–14. doi: 10.1021/ACS.JAFC.5B04543. 26785813

[2] Brookes G, Barfoot P. Environmental impacts of genetically modified (GM) crop use 1996–2018: impacts on pesticide use and carbon emissions. GM Crops Food. 2020;11(4):215–41. doi: 10.1080/21645698.2020.1773198. 32706316 PMC7518756

[3] Nerkar G, Devarumath S, Purankar M, Kumar A, Valarmathi R, Devarumath R, Appunu C. Advances in crop breeding through precision genome editing. Front Genet. 2022;13:880195. doi: 10.3389/FGENE.2022.880195. 35910205 PMC9329802

[4] Ahmad S, Tang L, Shahzad R, Mawia AM, Rao GS, Jamil S, Wei C, Sheng Z, Shao G, Wei X, et al. Crispr-based crop improvements: a way forward to achieve zero hunger. J Agric Food Chem. 2021;69(30):8307–23. doi: 10.1021/ACS.JAFC.1C02653. 34288688

[5] Bullion A, Malhotra B. Gene-edited crops market growth spurred by regulatory progress and approvals | S&P Global. [accessed 2025 04 2]. https://www.spglobal.com/commodity-insights/en/research-analytics/gene-edited-crops-market-growth-spurred-by-regulatory-progress.

[6] Gosal SS, Wani SH. Plant genetic transformation and transgenic crops: methods and applications. Biotech Crop Improv Transgenic Appr. 2018; 1–23. doi: 10.1007/978-3-319-90650-8_1.

[7] Devos Y, Demont M, Dillen K, Reheul D, Kaiser M, Sanvido O. Coexistence of genetically modified (GM) and non-GM crops in the European Union. A review. Agron Sustain Dev. 2009;29(1):11–30. doi: 10.1051/AGRO:2008051.

[8] Bruetschy C. The EU regulatory framework on genetically modified organisms (GMOs). Transgenic Res. 2019;28(2):169–74. doi: 10.1007/S11248-019-00149-Y. 31321701

[9] Turnbull C, Lillemo M, Hvoslef-Eide TAK. Global regulation of genetically modified crops amid the gene edited crop boom – a review. Front Plant Sci. 2021;12. doi: 10.3389/FPLS.2021.630396. PMC7943453 33719302

[10] European Commission. *Commission Regulation (EU)*. No 619/2011 of 24 June 2011 laying down the methods of sampling and analysis for the official control of feed as regards presence of genetically modified. Material for which an authorisation procedure is pending or the authorisation of which has expired text with EEA relevance. 2011 [accessed 2025 12 22]. https://eur-lex.europa.eu/eli/reg/2011/619/oj/eng.

[11] European Union Reference Laboratory for Genetically Modified Food and Feed (EURL GMFF). GMOMETHODS. [accessed 2023 10 9]. https://gmo-crl.jrc.ec.europa.eu/gmomethods/.

[12] Jacky L, Yurk D, Alvarado J, Belitz P, Fathe K, Macdonald C, Fraser S, Rajagopal A. Robust multichannel encoding for highly multiplexed quantitative PCR. Anal Chem. 2021;93(9):4208–16. doi: 10.1021/ACS.ANALCHEM.0C04626. 33631072

[13] Dobnik D, Štebih D, Blejec A, Morisset D, Žel J. Multiplex quantification of four DNA targets in one reaction with Bio-Rad droplet digital PCR system for GMO detection. Sci Rep. 2016;6(1):1–9. doi: 10.1038/srep35451. 27739510 PMC5064307

[14] Huber I, Block A, Sebah D, Debode F, Morisset D, Grohmann L, Berben G, Štebih D, Milavec M, Žel J, et al. Development and validation of duplex, triplex, and pentaplex real-time PCR screening assays for the detection of genetically modified organisms in food and feed. J Agric Food Chem. 2013;61(43):10293–301. doi: 10.1021/JF402448Y. 23971699

[15] Singh M, Pal D, Aminedi R, Singh AK. Multiplex real-time loop-mediated isothermal amplification (LAMP) based on the annealing curve analysis: toward an on-site multiplex detection of transgenic sequences in seeds and food products. J Agric Food Chem. 2024;72(31):17658–65. doi: 10.1021/ACS.JAFC.4C01803. 39044391

[16] Bogožalec Košir A, Spilsberg B, Holst-Jensen A, Žel J, Dobnik D. Development and inter-laboratory assessment of droplet digital PCR assays for multiplex quantification of 15 genetically modified soybean lines. Sci Rep. 2017;7(1):1–11. doi: 10.1038/s41598-017-09377-w. 28819142 PMC5561262

[17] Dobnik D, Spilsberg B, Bogožalec Košir A, Holst-Jensen A, Žel J. Multiplex quantification of 12 European Union authorized genetically modified maize lines with droplet digital polymerase chain reaction. Anal Chem. 2015;87(16):8218–26. doi: 10.1021/acs.analchem.5b01208. 26169291

[18] Pinheiro LB, Coleman VA, Hindson CM, Herrmann J, Hindson BJ, Bhat S, Emslie KR. Evaluation of a droplet digital polymerase chain reaction format for DNA copy number quantification. Anal Chem. 2011;84(2):1003–11. doi: 10.1021/AC202578X. 22122760 PMC3260738

[19] QIAGEN. Qiacuity nanoplates and accessories. [accessed 2025 12 22]. https://www.qiagen.com/us/products/instruments-and-automation/accessories/qiacuity-nanoplates-and-accessories.

[20] Trouchet A, Gines G, Benhaim L, Taly V. Digital PCR: from early developments to its future application in clinics. Lab Chip. 2025;25(16):3921–61. doi: 10.1039/d5lc00055f. 40686367 PMC12278255

[21] Demeke T, Dobnik D. Critical assessment of digital PCR for the detection and quantification of genetically modified organisms. Anal Bioanal Chem. 2018;410(17):4039–50. doi: 10.1007/S00216-018-1010-1. 29574561 PMC6010488

[22] Bogožalec Košir A, Muller S, Žel J, Milavec M, Mallory AC, Dobnik D. Fast and accurate multiplex identification and quantification of seven genetically modified soybean lines using six-color digital PCR. Foods. 2023;12(22):4156. doi: 10.3390/FOODS12224156. 38002213 PMC10670894

[23] Kudo E, Israelow B, Vogels CBF, Lu P, Wyllie AL, Tokuyama M, Venkataraman A, Brackney DE, Ott IM, Petrone ME, et al. Detection of SARS-CoV-2 RNA by multiplex RT-qPCR. PLOS Biol. 2020;18(10):e3000867. doi: 10.1371/JOURNAL.PBIO.3000867. 33027248 PMC7571696

[24] Bak A, Emerson JB. Multiplex quantitative PCR for single-reaction genetically modified (GM) plant detection and identification of false-positive GM plants linked to cauliflower mosaic virus (CaMV) infection. BMC Biotechnol. 2019;19(1):1–12. doi: 10.1186/s12896-019-0571-1. 31699075 PMC6836441

[25] de Korne-Elenbaas J, Caduff L, Lison A, McLeod R, Pitton M, Gan C, Julian TRD. Validation, and implementation of multiplex digital PCR assays for simultaneous quantification of multiple targets. Lett Appl Microbiol. 2025;78(1):137. doi: 10.1093/LAMBIO/OVAE137. 39701810

[26] Dobnik D, Spilsberg B, Bogožalec Košir A, Štebih D, Morisset D, Holst-Jensen A, Žel J. Multiplex droplet digital PCR protocols for quantification of GM maize events. In: Karlin-Neumann G, Bizouarn F, editors. Methods in molecular biology. Vol. 1768. New York (NY): Humana Press; 2018. p. 69–98. doi: 10.1007/978-1-4939-7778-9_5. 29717438

[27] European Commission. Regulation (EC) No 1831/2003 of the European Parliament and of the Council of 22 September 2003 on additives for use in animal nutrition. Off J Eur Union. 2003;268:29–43.

[28] Demeke T, Lee SJ, Eng M. Increasing the efficiency of canola and soybean GMO detection and quantification using multiplex droplet digital PCR. Biol (Basel). 2022;11(2):201. doi: 10.3390/BIOLOGY11020201/S1. PMC8869681 35205068

[29] Whale AS, Huggett JF, Tzonev S. Fundamentals of multiplexing with digital PCR. Biomol Detect Quantif. 2016;10:15–23. doi: 10.1016/J.BDQ.2016.05.002. 27990345 PMC5154634

[30] Bio-Rad Laboratories. Bio-Rad launches its QX ONE droplet digital PCR system to early access customers at AMP 2019 annual meeting & expo. [accessed 2023 10 10]. https://www.bio-rad.com/en-si/life-science-research/news/bio-rad-launches-its-qx-one-droplet-digital-pcr-system-early-access-customers-at-amp-2019-annual-meeting-expo?ID=Bio-Rad-Launches-Its_1573066471.

[31] Bio-Rad Laboratories. Qx600 droplet digital PCR system. [accessed 2025 01 24]. https://www.bio-rad.com/en-si/product/qx600-droplet-digital-pcr-system?ID=b07d12ac-0585-fc4c-a586-3ddf20d5c4a0.

[32] Thermo Fisher Scientific. QuantStudio absolute Q digital PCR system. [accessed 2023 10 13]. https://www.thermofisher.com/si/en/home/life-science/pcr/digital-pcr/quantstudio-absolute-q-system.html.

[33] QIAGEN. Qiacuity digital PCR system. [accessed 2023 10 13]. https://www.qiagen.com/us/products/instruments-and-automation/pcr-instruments/qiacuity-digital-pcr-system.

[34] Roche. Digital LightCycler® dPCR system. [accessed 2023 10 13]. https://sequencing.roche.com/us/en/products/group/digital-lightcycler-dpcr-system.html.

[35] Sniper Technologies. Sniper DQ24 digital PCR system. [accessed 2024 09 24]. https://www.sniper-tech.com/DQ24.html.

[36] Stilla Technologies. Nio^TM^+ dpcr. [accessed 2024 03 27]. https://www.stillatechnologies.com/multiplex-pcr/nio-dpcr/.

[37] Stilla Technologies. Naica® system: flexible multiplex digital PCR solution. accessed 2023 10 20. https://www.stillatechnologies.com/.

[38] European Commission. Genetically modified organisms. EU register of authorised GMOs. [accessed 2023 10 13]. https://webgate.ec.europa.eu/dyna2/gm-register/.

[39] Vallone PM, Butler JM. Autodimer: a screening tool for primer-dimer and hairpin structures. Biotechniques. 2004;37(2):226–31. doi: 10.2144/04372ST03. 15335214

[40] Wang K, Li H, Xu Y, Shao Q, Yi J, Wang R, Cai W, Hang X, Zhang C, Cai H, et al. Mfeprimer-3.0: Quality control for PCR primers. Nucleic Acids Res. 2019;47(W1):W610–13. doi: 10.1093/NAR/GKZ351. 31066442 PMC6602485

[41] Qu W, Zhou Y, Zhang Y, Lu Y, Wang X, Zhao D, Yang Y, Zhang C. Mfeprimer-2.0: a fast thermodynamics-based program for checking PCR primer specificity. Nucleic Acids Res. 2012;40(W1):W205–08. doi: 10.1093/NAR/GKS552. 22689644 PMC3394324

[42] Corbisier P, Buttinger G, Savini C, Sacco MG, Gatto F, Emons H. Expression of GM content in mass fraction from digital PCR data. Food Control. 2022;133:108626. doi: 10.1016/J.FOODCONT.2021.108626. 35241875 PMC8756621

[43] European Commission Joint research centre Directorate F - Food & Feed Compliance (EC JRC Directorate F). Conversion factors (CF) for certified references materials (CRM) (version 13 - 09/12/2025). 2025.

[44] European Network of GMO Laboratories (ENGL). 2015. Definition of minimum performance requirements for analytical methods of GMO testing. European Commission Joint research centre Institute for Health and Consumer Protection.

[45] Demeke T, Eng M. Detection of soybean GMO events using two multiplex droplet digital PCR assays. J AOAC Int. 2024;108(1):23–28. doi: 10.1093/JAOACINT/QSAE082. PMC11761968 39475430

[46] Savini C, Sacco MG, Mazzara M, Vincent U. Event-specific method for the quantification of soybean event MON 94313 using real-time PCR. 2024 [accessed 2025 04 7]. https://joint-research-centre.ec.europa.eu.

[47] Gmo application details. [accessed 2025 04 9]. https://euginius.eu/euginius/pages/application_detail.jsf?application=-1964886332877943212.

[48] Release note: Qiacuity software suite (v3.0) - Qiagen. [accessed 2025 04 9]. https://www.qiagen.com/us/resources/resourcedetail?id=2a70b193-7ee1-47f7-a5d4-927763dad84b&lang=en.

[49] Bussmann M, Hesse M, Karalay O, Nash R, Missel A. Impact of template addition volume and analyzed volume on digital PCR sensitivity. 2022 [accessed 2024 08 29]. https://www.qiagen.com/us/resources/resourcedetail?id=625d0d8b-278b-4796-bff4-2cb04533d712&lang=en.

[50] Lievens A, Jacchia S, Kagkli D, Savini C, Querci M. Measuring digital PCR quality: performance parameters and their optimization. PLOS ONE. 2016;11(5):e0153317. doi: 10.1371/JOURNAL.PONE.0153317. 27149415 PMC4858304

[51] Pavšič J, Žel J, Milavec M. Assessment of the real-time PCR and different digital PCR platforms for DNA quantification. Anal Bioanal Chem. 2016;408(1):107–21. doi: 10.1007/S00216-015-9107-2. 26521179 PMC4706846

