# Supplementary material for: Simple, fast, reliable: multiplex digital PCR quantification of 19 genetically modified soybean events
Source: GM Crops Food. 2026 Feb 27;17(1):2635816. doi: 10.1080/21645698.2026.2635816 (PMC12959185; doi:10.1080/21645698.2026.2635816)
Supplement: Supplementary information 1_Tables and figures clean.docx [file KGMC_A_2635816_SM9268.docx]

SuppLEMENTARY infromation

Simple, Fast, Reliable: Multiplex Digital PCR Quantification of 19 Genetically Modified Soybean Events

Amadej Jelenčič^a,b^, Dejan Štebih^a^, Tina Demšar^a^ and David Dobnik^a^

^a^Department of Biotechnology and Systems Biology, National Institute of Biology, Večna pot 121, SI-1000 Ljubljana, Slovenia

^b^Jozef Stefan International Postgraduate School, Jamova 39, SI-1000 Ljubljana, Slovenija

KEYWORDS: genetically modified (GM) crops, transgenic, soybean, Glycine max, quantification, digital PCR (dPCR), multiplex, multi-target

Table S1. Primers and Probes Used in the 5-Plex Assays, Their Final Concentrations in The Reaction, and the Lengths of the Amplified Sequences

| **Event/target** | **Primer/probe** | **Sequence (5’-sequence-3’)** | **Final Concentration in the reaction [nmol/L]** | **Amplicon length (bp)** |
| --- | --- | --- | --- | --- |
| DBN-09004-6 | F | GCCGTATCCGCAATGTGTTA | 600 | 89 |
|  | R | GCTCCATAAACGTGTGCTTTCA | 600 |  |
|  | P | FAM-TTGTTTACAACTCTGTGACCC-MGBNFQ | 200 |  |
| GMB151 | F | TCAAATCAACATGGGTGACTAGAAA | 400 | 84 |
|  | R | CATTGTGCTGAATAGGTTTATAGCTATGAT | 400 |  |
|  | P | HEX-CAGTACTGG/ZEN/GCCCTTGTGGCGCT-IAbFQ | 200 |  |
| SYHT0H2 | F | GGGAATTGGGTACCATGCC | 600 | 88 |
|  | R | TGTGTGCCATTGGTTTAGGGT | 600 |  |
|  | P | TAMRA-CCAGCATGGCCGTATCCGCAA-BHQ2 | 200 |  |
| MON87751 | F | CTAAATTGCTCTTTGGAGTTTATTTTGTAG | 500 | 87 |
|  | R | GGCCTAACTTTTGGTGTGATGATG | 500 |  |
|  | P | TexRd-TGACTGGAGATCTCCAAAGTGAGGGGAAA-IAbRQ | 300 |  |
| Le1 | F | CCAGCTTCGCCGCTTCCTTC | 650 | 74 |
|  | R | GAAGGCAAGCCCATCTGCAAGCC | 650 |  |
|  | P | Cy5-CTTCACCTT/TAO/CTATGCCCCTGACAC-IAbRQ | 180 |  |
| 305423 | F | CGTGTTCTCTTTTTGGCTAGC | 800 | 93 |
|  | R | GTGACCAATGAATACATAACACAAACTA | 500 |  |
|  | P | FAM-TGACACAAA/ZEN/TGATTTTCATACAAAAGTCGAGA-IAbFQ | 220 |  |
| BPS-CV127-9 | F | AACAGAAGTTTCCGTTGAGCTTTAAGAC | 400 | 88 |
|  | R | CATTCGTAGCTCGGATCGTGTAC | 400 |  |
|  | P | HEX-TTTGGGGAA/ZEN/GCTGTCCCATGCCC-IAbFQ | 100 |  |
| Continued on the next page. | | | | |
| Table S1, continued | | | | |
| **Event/target** | **Primer/probe** | **Sequence (5’-sequence-3’)** | **Final Concentration in the reaction [nmol/L]** | **Amplicon length (bp)** |
| MON87769 | F | CATACTCATTGCTGATCCATGTAGATT | 600 | 87 |
|  | R | GCAAGTTGCTCGTGAAGTTTTG | 600 |  |
|  | P | TAMRA-CCCGGACATGAAGCCATTTACAATTGAC-BHQ2 | 200 |  |
| MON87701 | F | TGGTGATATGAAGATACATGCTTAGCAT | 600 | 81 |
|  | R | CGTTTCCCGCCTTCAGTTTAAA | 600 |  |
|  | P | TexRd-TCAGTGTTTGACACACACACTAAGCGTGCC-IAbRQ | 250 |  |
| MON87708 | F | TCATACTCATTGCTGATCCATGTAG | 300 | 91 |
|  | R | AGAACAAATTAACGAAAAGACAGAACG | 300 |  |
|  | P | Cy5-TCCCGGACT/TAO/TTAGCTCAAAATGCATGTA-IAbRQ | 150 |  |
| 40-3-2 | F | TTCATTCAAAATAAGATCATACATACAGGTT | 600 | 84 |
|  | R | GGCATTTGTAGGAGCCACCTT | 600 |  |
|  | P | FAM-CCTTTTCCATTTGGG-MGBNFQ | 200 |  |
| A5547-127 | F | GCTATTTGGTGGCATTTTTCCA | 400 | 75 |
|  | R | CACTGCGGCCAACTTACTTCT | 400 |  |
|  | P | HEX-CCGCAATGT/ZEN/CATACCGTCATCGTTGT-IAbFQ | 200 |  |
| A2704-12 | F | GCAAAAAAGCGGTTAGCTCCT | 400 | 64 |
|  | R | ATTCAGGCTGCGCAACTGTT | 400 |  |
|  | P | TAMRA-CGGTCCTCCGATCGCCCTTCC-BHQ2 | 200 |  |
| MON89788 | F | TCCCGCTCTAGCGCTTCAAT | 600 | 139 |
|  | R | TCGAGCAGGACCTGCAGAA | 600 |  |
|  | P | TexRd-CTGAAGGCGGGAAACGACAATCTG-IAbRQ | 200 |  |
| MON87705 | F | TTCCCGGACATGAAGCCATTTAC | 450 | 86 |
|  | R | ACAACGGTGCCTTGGCCCAAAG | 450 |  |
|  | P | Cy5-AAGAGACTC/TAO/AGGGTGTTGTTATCACTGCGG-IAbRQ | 250 |  |
| FG72 | F | AGATTTGATCGGGCTGCAGG | 400 | 70 |
|  | R | GCACGTATTGATGACCGCATTA | 400 |  |
|  | P | FAM-AATGTGGTTCATCCGTCTT-MGBNFQ | 200 |  |
| DAS-68416-4 | F | GTACATTAAAAACGTCCGCAATGTGT | 550 | 130 |
|  | R | GTTTAAGAATTAGTTCTTACAGTTTATTGTTAG | 550 |  |
|  | P | HEX-TTAAGTTGT/ZEN/CTAAGCGTCAATA-IAbFQ | 150 |  |
| 356043 | F | GTCGAATAGGCTAGGTTTACGAAAAA | 750 | 99 |
|  | R | TTTGATATTCTTGGAGTAGACGAGAGTGT | 750 |  |
|  | P | TAMRA-CTCTAGAGATCCGTCAACATGGTGGAGCAC-BHQ2 | 200 |  |
| Continued on the next page. | | | | |
|  | |  |  | |
|  |  |  |  |  |
| Table S1, continued | | | | |
| **Event/target** | **Primer/probe** | **Sequence (5’-sequence-3’)** | **Final Concentration in the reaction [nmol/L]** | **Amplicon length (bp)** |
| DAS-81419-2 | F | TCTAGCTATATTTAGCACTTGATATTCAT | 400 | 105 |
|  | R | GCTTCAAGATCCCAACTTGCG | 400 |  |
|  | P | TexRd-CGGTCCTCCGATCGCCCTTCC-IAbRQ | 120 |  |
| DAS-44406-6 | F | TTATTGTTCTTGTTGTTTCCTCTTTAGG | 300 | 99 |
|  | R | CCTCAATTGCGAGCTTTCTAATTT | 300 |  |
|  | P | Cy5-ATTCGGACC/TAO/TCCATGATGACCTTACCGTT-IAbRQ | 180 |  |

^a^The abbreviations for used fluorescent dyes and quenchers are shown with probe sequences on 5’ and 3’ ends, respectively. ZEN and TAO internal quenchers are included in the sequences of double-quenched probes. Some probes include a minor groove binder (MGB) moiety. FAM – fluorescein, HEX – hexachlorofluorescein, TAMRA – 5-carboxytetramethylrhodamine, TexRd – Texas Red, Cy5 - Cyanine5, NFQ – nonfluorescent quencher, BHQ2 – Black Hole Quencher 2, IAbFQ – Iowa Bloack Fluorescence Quencher, IAbRQ – Iowa Black RQ.

Table S2. The EURL Reference Methods Used in the 5-plex Assays.

| **Event/gene** | **Unique identifier** | **Legal status** | **Assay** | **EURL-GMFF method reference** |
| --- | --- | --- | --- | --- |
| DBN-09004-6 | DBN-Ø9ØØ4-6 | Pending | 5-plex 1 | [QT-EVE-GM-019](https://gmo-crl.jrc.ec.europa.eu/gmomethods/entry?db=gmometh&nr=140&q=id%3aQT-eve-gm*) |
| GMB151 | BCS-GM151-6 | Authorized | 5-plex 1 | [QT-EVE-GM-018](https://gmo-crl.jrc.ec.europa.eu/gmomethods/entry?db=gmometh&nr=137&q=id%3aQT-eve-gm*) |
| SYHT0H2 | SYN-ØØØH2-5 | Authorized | 5-plex 1 | [QT-EVE-GM-017](https://gmo-crl.jrc.ec.europa.eu/gmomethods/entry?db=gmometh&nr=136&q=id%3aQT-eve-gm*) |
| MON87751 | MON-87751-7 | Authorized | 5-plex 1 | [QT-EVE-GM-016](https://gmo-crl.jrc.ec.europa.eu/gmomethods/entry?db=gmometh&nr=135&q=id%3aQT-eve-gm*) |
| *Le1* | / | / | 5-plex 1 | [QT-EVE-GM-001](https://gmo-crl.jrc.ec.europa.eu/gmomethods/entry?db=gmometh&nr=120&q=id%3aQT-eve-gm*) |
| 305423 | DP-3Ø5423-1 | Authorized | 5-plex 2 | [QT-EVE-GM-008](https://gmo-crl.jrc.ec.europa.eu/gmomethods/entry?db=gmometh&nr=127&q=id%3aQT-eve-gm*) |
| BPS-CV127-9 | BPS-CV127-9 | Authorized | 5-plex 2 | [QT-EVE-GM-011](https://gmo-crl.jrc.ec.europa.eu/gmomethods/entry?db=gmometh&nr=130&q=id%3aQT-eve-gm*) |
| MON87769 | MON-87769-7 | Authorized | 5-plex 2 | [QT-EVE-GM-002](https://gmo-crl.jrc.ec.europa.eu/gmomethods/entry?db=gmometh&nr=122&q=id%3aQT-eve-gm*) |
| MON87701 | MON-877Ø1-2 | Authorized | 5-plex 2 | [QT-EVE-GM-010](https://gmo-crl.jrc.ec.europa.eu/gmomethods/entry?db=gmometh&nr=129&q=id%3aQT-eve-gm*) |
| MON87708 | MON-877Ø8-9 | Authorized | 5-plex 2 | [QT-EVE-GM-012](https://gmo-crl.jrc.ec.europa.eu/gmomethods/entry?db=gmometh&nr=131&q=id%3aQT-eve-gm*) |
| 40-3-2 | MON-Ø4Ø32-6 | Authorized | 5-plex 3 | [QT-EVE-GM-005](https://gmo-crl.jrc.ec.europa.eu/gmomethods/entry?db=gmometh&nr=125&q=id%3aQT-eve-gm*) |
| A5547-127 | ACS-GMØØ6-4 | Authorized | 5-plex 3 | [QT-EVE-GM-007](https://gmo-crl.jrc.ec.europa.eu/gmomethods/entry?db=gmometh&nr=126&q=id%3aQT-eve-gm*) |
| A2704-12 | ACS-GMØØ5-3 | Authorized | 5-plex 3 | [QT-EVE-GM-004](https://gmo-crl.jrc.ec.europa.eu/gmomethods/entry?db=gmometh&nr=121&q=id%3aQT-eve-gm*) |
| MON89788 | MON-89788-1 | Authorized | 5-plex 3 | [QT-EVE-GM-006](https://gmo-crl.jrc.ec.europa.eu/gmomethods/entry?db=gmometh&nr=124&q=id%3aQT-eve-gm*) |
| MON87705 | MON-877Ø5-6 | Authorized | 5-plex 3 | [QT-EVE-GM-003](https://gmo-crl.jrc.ec.europa.eu/gmomethods/entry?db=gmometh&nr=123&q=id%3aQT-eve-gm*) |
| FG72 | MST-FGØ72-2 | Authorized | 5-plex 4 | [QT-EVE-GM-001](https://gmo-crl.jrc.ec.europa.eu/gmomethods/entry?db=gmometh&nr=120&q=id%3aQT-eve-gm*) |
| DAS-68416-4 | DAS-68416-4 | Authorized | 5-plex 4 | [QT-EVE-GM-013](https://gmo-crl.jrc.ec.europa.eu/gmomethods/entry?db=gmometh&nr=132&q=id%3aQT-eve-gm*) |
| 356043 | DP-356Ø43-5 | Expired | 5-plex 4 | [QT-EVE-GM-009](https://gmo-crl.jrc.ec.europa.eu/gmomethods/entry?db=gmometh&nr=128&q=id%3aQT-eve-gm*) |
| DAS-81419-2 | DAS-81419-2 | Authorized | 5-plex 4 | [QT-EVE-GM-014](https://gmo-crl.jrc.ec.europa.eu/gmomethods/entry?db=gmometh&nr=134&q=id%3aQT-eve-gm*) |
| DAS-44406-6 | DAS-444Ø6-6 | Authorized | 5-plex 4 | [QT-EVE-GM-015](https://gmo-crl.jrc.ec.europa.eu/gmomethods/entry?db=gmometh&nr=133&q=id%3aQT-eve-gm*) |

Table S3. Comparison Between Multiplex and Simplex Assays on Single Event DNA Samples

| **GM event/gene** | **Cp/rxn simplex^a^** | **Cp/rxn 5-plex^a^** | **Bias 5-plex/simplex (%)** |
| --- | --- | --- | --- |
| DBN-09004-6 | 76486 | 73466 | -4.0 |
| GMB151 | 44520 | 45616 | 2.5 |
| SYHT0H2 | 60692 | 60561 | -0.22 |
| MON87751 | 88165 | 83136 | -5.7 |
| *Le1^b^* | 74550 | 78757 | 5.6 |
| 305423 | 1990 | 1741 | 4.0 |
| BPS-CV127-9 | 301723 | 297668 | -1.3 |
| MON87769 | 79136 | 75926 | -4.0 |
| MON87701 | 68771 | 68713 | -0.1 |
| MON87708 | 62490 | 64975 | 4.0 |
| 40-3-2 | 44130 | 47862 | 8.5 |
| A5547-127 | 23672 | 24115 | 1.9 |
| A2704-12 | 75410 | 67982 | -9.9 |
| MON89788 | 44401 | 46802 | 5.4 |
| MON87705 | 91555 | 95113 | 3.9 |
| FG72 | 34005 | 37493 | 10.3 |
| DAS-68416-4 | 7806 | 8284 | 6.1 |
| 356043 | 6616 | 5897 | -10.9 |
| DAS-81419-2 | 70557 | 61575 | -12.7 |
| DAS-44406-6 | 52767 | 45287 | -14.2 |

^a^The experimental results are presented as the mean target copy numbers per analyzed reaction volume (cp/rxn), normalized to undiluted sample conditions, based on 4 total technical replicates across 2 sample dilutions.

^b^Bias for *Le1* was <25% for all tested samples (dilutions of individual GM soybean event certified reference material (CRM) DNA). Here, results for sample containing DNA of event DBN-9004-6 are shown.

Table S4. Comparison Between Multiplex and Simplex Assays on DNA Mixtures of Events Quantified by the Respective Assays

| **GM event/gene** | **Cp/rxn simplex^a,b^** | **Cp/rxn 5-plex^a^** | **Bias 5-plex/simplex (%)** |
| --- | --- | --- | --- |
| DBN-09004-6 | 79256 | 79459 | 0.3 |
| GMB151 | 43612 | 48859 | 12.0 |
| SYHT0H2 | 65463 | 62690 | -4.2 |
| MON87751 | 91138 | 91233 | 0.1 |
| *Le1^c^* | 263880 | 281378 | 6.6 |
| 305423 | 8091 | 7924 | -2.0 |
| BPS-CV127-9 | 299152 | 313229 | 4.7 |
| MON87769 | 72230^d^ | 77644 | 7.5 |
| MON87701 | 71212 | 67301 | -5.5 |
| MON87708 | 33321 | 27177 | -18.4 |
| 40-3-2 | 34447 | 37889 | 10.0 |
| A5547-127 | 48555^d^ | 54285 | 11.8 |
| A2704-12 | 69034 | 74938 | 8.6 |
| MON89788 | 64809 | 74115 | 14.4 |
| MON87705 | 87470 | 80338 | -8.2 |
| FG72 | 56817 | 53796 | -5.3 |
| DAS-68416-4 | 9356 | 10084 | 7.8 |
| 356043 | 6123 | 6949 | 13.5 |
| DAS-81419-2 | 64464 | 69839 | 8.3 |
| DAS-44406-6 | 49632 | 52999 | 6.8 |

^a^The experimental results are presented as the mean target copy numbers per analyzed reaction volume (cp/rxn), normalized to undiluted sample conditions, based on up to 2 technical replicates.

^b^Some replicates were rejected from analysis by the software due to an imaging issue.

^c^Bias for *Le1* was <25% for all tested samples (Soybean DNA mixtures 1-4 corresponding to GM events quantified 5-plexes 1-4). Here, results for DNA mix 1 are shown, containing DNA of events DBN-09004-6, GMB151, SYHT0H2, and MON87751.

^d^Result from only one technical replicate.

Table S5. Trueness of 5-plex Assays

| **GM event** | **GM% certified value^a^** | **GM% 5-plex^b^** | **Bias 5-plex/assigned (%)** |
| --- | --- | --- | --- |
| DBN-09004-6 | 100.0 | 93.3 | -6.7 |
| GMB151 | 100.0 | 108.0 | 8.0 |
| SYHT0H2 | 100.0 | 103.0 | 3.0 |
| MON87751 | 100.0 | 100.2 | 0.2 |
| 305423 | 10.0 | 9.52 | -4.8 |
| BPS-CV127-9 | 100.0 | 90.1 | -9.91 |
| MON87769 | 100.0 | 96.7 | -3.3 |
| MON87701 | 99.2 | 88.0 | -11.3 |
| MON87708 | 99.9 | 97.3 | -2.6 |
| 40-3-2 | > 98.5 | 100.5 | 4.0 |
| A5547-127 | 100.0 | 91.1 | -8.9 |
| A2704-12 | 100.0 | 95.4 | -4.6 |
| MON89788 | 100.0 | 85.1 | -14.9 |
| MON87705 | 100.0 | 95.4 | -4.6 |
| FG72 | 100.0 | 134.4^c^ | 34.4^c^ |
| DAS-68416-4 | 10.0 | 11.2 | -11.6 |
| 356043 | 10.0 | 9.4 | -5.8 |
| DAS-81419-2 | > 98.6 | 90.2 | -8.5 |
| DAS-44406-6 | > 98.6 | 92.5 | -6.2 |

^a^Certified values are the official GM% values provided with the respective certified reference materials (CRMs; see main text Materials and methods section for a list of CRMs used).

^b^The experimental results are presented as the mean GM event percentages obtained from 4 total technical replicates across 2 sample dilutions.

^c^The GM% value was obtained by utilizing the CF value of 1.00. The CF expanded uncertainty in 0.10. If we calculate using the CF value at the upper bound of this uncertainty (1.10), we obtain the GM% value of 122.2%, which would mean 22.2% bias from the certified value­—under the threshold of 25%.

Table S6. Dynamic Range in copies per reaction (cp/rxn) for 5-plex 1 with experimental results presented as mean of 16 technical replicates with the corresponding coefficient of variation (CV).

| GM event/gene | Cp/rxn | CV (%) |
| --- | --- | --- |
| DBN-09004-6 | 2580 | 4,9 |
|  | 1374 | 5,1 |
|  | 354 | 6,5 |
|  | 74 | 13,1 |
|  | 28 | 17,9 |
|  | 1 | 90,2 |
|  | 0 | 400,0 |
| GMB151 | 2923 | 3,7 |
|  | 1572 | 5,0 |
|  | 446 | 9,0 |
|  | 93 | 12,6 |
|  | 36 | 20,6 |
|  | 2 | 69,9 |
|  | 0 | 290,1 |
| SYHT0H2 | 2582 | 8,1 |
|  | 1438 | 5,7 |
|  | 389 | 8,7 |
|  | 80 | 13,2 |
|  | 34 | 18,7 |
|  | 2 | 68,7 |
|  | 0 | 192,0 |
| MON87751 | 2779 | 3,8 |
|  | 1442 | 4,7 |
|  | 388 | 10,2 |
|  | 84 | 12,1 |
|  | 35 | 19,0 |
|  | 1 | 72,2 |
|  | 0 | 179,0 |
| *Le1* | /^a,b^ | / |
|  | 59360^a^ | 8,0 |
|  | 16881 | 5,2 |
|  | 3533 | 4,7 |
|  | 1417 | 7,4 |
|  | 70 | 13,9 |
|  | **8** | **36,8** |

Underlined number – lower bound of tested range/limit of quantification (LOQ).

Red number – at least 1 technical replicate negative.

Bold number- result considered as LOD for *Le1*.

^a^Quantification not accurate (<20 partitions negative).

^b^Some wells reached saturation (all positive partitions).

Table S7. Dynamic Range in copies per reaction (cp/rxn) for 5-plex 2 with experimental results presented as mean of 16 technical replicates with the corresponding coefficient of variation (CV).

| GM event | Cp/rxn | CV (%) |
| --- | --- | --- |
| 305423 | 2817 | 6,5 |
|  | 1468 | 5,0 |
|  | 328 | 6,8 |
|  | 72 | 10 |
|  | 27 | 22,4 |
|  | 1 | 109,0 |
|  | 0 | 192,5 |
| BPS-CV127-9 | 2756 | 5,6 |
|  | 1389 | 5,1 |
|  | 428 | 5,4 |
|  | 83 | 10 |
|  | 33 | 23,9 |
|  | 1 | 71,5 |
|  | 0 | 231,0 |
| MON87769 | 2600 | 6,4 |
|  | 1394 | 5,4 |
|  | 339 | 6,0 |
|  | 73 | 13,0 |
|  | 28 | 18,4 |
|  | 1 | 67,0 |
|  | 0 | 215,0 |
| MON87701 | 2946 | 7,0 |
|  | 1479 | 4,0 |
|  | 371 | 5,4 |
|  | 85 | 13,6 |
|  | 31 | 15,7 |
|  | 2 | 83,7 |
|  | 0 | 192,8 |
| MON87708 | 2777 | 5,7 |
|  | 1411 | 4,7 |
|  | 371 | 7,4 |
|  | 85 | 14,0 |
|  | 31 | 21,8 |
|  | 2 | 72,6 |
|  | 0 | 273,3 |

Underlined number – lower bound of tested range/limit of quantification (LOQ).

Red number – at least 1 technical replicate negative.

Table S8. Dynamic Range in copies per reaction (cp/rxn) for 5-plex 3 with experimental results presented as mean of 16 technical replicates with the corresponding coefficient of variation (CV).

| GM event | Cp/rxn | CV (%) |
| --- | --- | --- |
| 40-3-2 | 3249 | 3,8 |
|  | 1660 | 2,8 |
|  | 425 | 5,3 |
|  | 97 | 12,4 |
|  | 36 | 19,7 |
|  | 2 | 57,2 |
|  | 1 | 126,7 |
| A5547-127 | 3003 | 3,2 |
|  | 1494 | 3,7 |
|  | 336 | 6,2 |
|  | 91 | 12,3 |
|  | 34 | 18,4 |
|  | 2 | 67,5 |
|  | 0 | 273,3 |
| A2704-12 | 2956 | 3,9 |
|  | 1474 | 3,2 |
|  | 323 | 7,7 |
|  | 83 | 11,0 |
|  | 31 | 17,8 |
|  | 2 | 106,1 |
|  | 0 | 215,0 |
| MON89788 | 2764 | 3,2 |
|  | 1379 | 3,8 |
|  | 415 | 6,8 |
|  | 88 | 10,1 |
|  | 35 | 20,1 |
|  | 2 | 56,1 |
|  | 0 | 273,3 |
| MON87705 | 2797 | 4,5 |
|  | 1439 | 5,0 |
|  | 357 | 6,4 |
|  | 70 | 11,3 |
|  | 27 | 17,5 |
|  | 1 | 74,5 |
|  | 0 | 215,0 |

Underlined number – lower bound of tested range/limit of quantification (LOQ).

Red number – at least 1 technical replicate negative.

Table S9. Dynamic Range in copies per reaction (cp/rxn) for 5-plex 4 with experimental results presented as mean of 16 technical replicates with the corresponding coefficient of variation (CV).

| GM event | Cp/rxn | CV (%) |
| --- | --- | --- |
| FG72 | 2839 | 2,1 |
|  | 1425 | 3,6 |
|  | 440 | 5,3 |
|  | 98 | 14,5 |
|  | 36 | 13,7 |
|  | 2 | 80,3 |
|  | 0 | 178,9 |
| DAS-68416-4 | 2734 | 3,0 |
|  | 1367 | 3,4 |
|  | 377 | 5,7 |
|  | 83 | 11,2 |
|  | 30 | 23,9 |
|  | 1 | 87,9 |
|  | 0 | 273,3 |
| 356043 | 2640 | 2,7 |
|  | 1343 | 4,3 |
|  | 366 | 7,4 |
|  | 75 | 13,9 |
|  | 29 | 17,6 |
|  | 1 | 115,7 |
|  | 0 | 273,3 |
| DAS-81419-2 | 2704 | 2,7 |
|  | 1336 | 3,5 |
|  | 338 | 7,3 |
|  | 77 | 11,5 |
|  | 28 | 15,8 |
|  | 1 | 60,7 |
|  | 0 | 273,3 |
| DAS-44406-6 | 2795 | 5,0 |
|  | 1406 | 5,2 |
|  | 344 | 6,5 |
|  | 77 | 7,1 |
|  | 31 | 13,7 |
|  | 1 | 92,4 |
|  | 0 | - |

Underlined number – lower bound of tested range/limit of quantification (LOQ).

Red number – at least 1 technical replicate negative.

Table S10. Limits of Detection (LOD) in copies per reaction (cp/rxn) for targets detected by 5-plexes 1-4 with experimental results presented as mean of 16 technical replicates.

| **GM event/gene** | **Cp/rxn^a^** |
| --- | --- |
| DBN9004 | 15 |
| GMB151 | 19 |
| SYHT0H2 | 18 |
| MON87751 | 17 |
| *Le1* | 8 |
| DP305423 | 13 |
| CV127 | 17 |
| MON87769 | 16 |
| MON87701 | 16 |
| MON87708 | 14 |
| MON40-3-2 | 16 |
| A5547 | 15 |
| A2704 | 14 |
| MON89788 | 17 |
| MON87705 | 14 |
| FG72 | 19 |
| DAS68416 | 16 |
| DP356043 | 15 |
| DAS81419 | 16 |
| DAS44406 | 16 |

Table S11. Asymmetric Limits of Quantification (LOQ) in copies per reaction (cp/rxn) for 5-plex 1 with experimental results presented as mean of 9 technical replicates with the corresponding coefficient of variation (CV).

| GM event | Cp/rxn | CV (%) |
| --- | --- | --- |
| DBN-09004-6 | 47 | 6.7 |
|  | 34 | 16.2 |
|  | 17 | 26.9 |
|  | **11** | **42.7** |
|  | 4 | 35.5 |
| GMB151 | 45 | 17.6 |
|  | 34 | 22.6 |
|  | 16 | 31.7 |
|  | **7** | **36.6** |
|  | 4 | 57.5 |
| SYHT0H2 | 45 | 22.1 |
|  | 42 | 24.9 |
|  | 20 | 27.6 |
|  | 9 | 18.6 |
|  | **5** | **41.5** |
| MON87751 | 79 | 8.1 |
|  | 64 | 11.7 |
|  | 35 | 15.4 |
|  | 16 | 23.4 |
|  | **7** | **25.5** |
| *Le1^a^* | 99 | 15.2 |
|  | 39 | 18.0 |
|  | 19 | 25.9 |

Underlined number – result considered as asymmetric LOQ (symmetric for *Le1*).

Bold number – result considered as asymmetric LOD.

Red number – at least one technical replicate negative.

^a^Determination of LOQ for *Le1* was performed in symmetric conditions by testing dilutions of a mixture of 4 GM soybean events (DBN-09004-6, GMB151, SYHT0H2, and MON87751) in 25 technical replicates.

Table S12. Asymmetric Limits of Quantification (LOQ) in copies per reaction (cp/rxn) for 5-plex 2 with experimental results presented as mean of 9 technical replicates with the corresponding coefficient of variation (CV).

| GM event/gene | Cp/rxn | CV (%) |
| --- | --- | --- |
| 305423 | 52 | 9.0 |
|  | 42 | 8.3 |
|  | 21 | 21.1 |
|  | **16** | **23.9** |
|  | 5 | 62.3 |
| BPS-CV127-9 | 48 | 14.7 |
|  | 38 | 9.0 |
|  | 20 | 20.7 |
|  | **9** | **25.8** |
|  | 4 | 36.5 |
| MON87769 | 48 | 20.6 |
|  | 36 | 13.0 |
|  | 18 | 26.5 |
|  | **9** | **51.8** |
|  | 5 | 35.5 |
| MON87701 | 48 | 20.6 |
|  | 42 | 24.1 |
|  | 16 | 28.2 |
|  | **9** | **48.6** |
|  | 3 | 46.9 |
| MON87708 | 41 | 23.1 |
|  | 39 | 15.3 |
|  | 18 | 18.3 |
|  | **8** | **33.5** |
|  | 4 | 57.7 |

Underlined number – result considered as asymmetric LOQ.

Bold number – result considered as asymmetric LOD.

Red number – at least one technical replicate negative.

Table S13. Asymmetric limits of quantification (LOQ) in copies per reaction (cp/rxn) for 5-plex 3 with experimental results presented as mean of 9 technical replicates with the corresponding coefficient of variation (CV).

| GM event/gene | Cp/rxn | CV (%) |
| --- | --- | --- |
| 40-3-2 | 47^a^ | 18.7 |
|  | 44 | 9.6 |
|  | 18 | 33.5 |
|  | 12 | 34.8 |
|  | **5** | **32.9** |
| A5547-127 | 57^a^ | 11.7 |
|  | 43 | 10.4 |
|  | 23 | 21.9 |
|  | 11 | 29.3 |
|  | **6** | **45.0** |
| A2704-12 | 52 | 14.7 |
|  | 39 | 23.1 |
|  | 20 | 35.4 |
|  | 10 | 35.7 |
|  | **5** | **45.1** |
| MON89788 | 68 | 15.6 |
|  | 50 | 14.6 |
|  | 27 | 21.6 |
|  | 11 | 30.4 |
|  | **5** | **29.7** |
| MON87705 | 58 | 14.4 |
|  | 43 | 12.7 |
|  | 25 | 19.4 |
|  | **10** | **33.8** |
|  | 4 | 73.3 |

Underlined number – result considered as asymmetric LOQ.

Bold number – result considered as asymmetric LOD.

Red number – at least one technical replicate negative.

^a^One technical replicate was excluded from analysis by the QIAcuity software.

Table S14: Asymmetric limits of detection (LOD) and limits of quantification (LOQ) in copies per reaction (cp/rxn) for 5-plex 4 with experimental results presented as mean of 9 technical replicates with the corresponding coefficient of variation (CV).

| GM event/gene | Cp/rxn | CV (%) |
| --- | --- | --- |
| FG72 | 35 | 14.2 |
|  | 29 | 8.5 |
|  | **15** | **29.8** |
|  | 8 | 36.8 |
|  | 3 | 50.0 |
| DAS-68416-4 | 49 | 13.7 |
|  | 39 | 18.5 |
|  | 21 | 23.7 |
|  | **10** | **38.3** |
|  | 5^a^ | 43.2 |
| 356043 | 49 | 7.9 |
|  | 39 | 24.9 |
|  | 22 | 27.9 |
|  | **8^a^** | **42.0** |
|  | 5 | 50.5 |
| DAS-81419-2 | 45 | 22.1 |
|  | 33 | 26.5 |
|  | 20 | 30.6 |
|  | 8^a^ | 22.6 |
|  | **6** | **19.7** |
| DAS-44406-6 | 45 | 8.2 |
|  | 38 | 19.7 |
|  | 18 | 22.9 |
|  | **9** | **35.0** |
|  | 5 | 49.6 |

Underlined number – result considered as asymmetric LOQ.

Bold number – result considered as asymmetric LOD.

Red number – at least one technical replicate negative.

^a^One technical replicate was excluded from analysis as an outlier, determined by the GraphPad online outlier calculator with default settings (https://www.graphpad.com/quickcalcs/grubbs1/).

Table S15: Robustness of 5-plex dPCR assays.

| GM event/gene | Normal conditions | T -1°C | | T +1°C | | PP -10% | | PP +10% | | CV (%)^c^ |
| --- | --- | --- | --- | --- | --- | --- | --- | --- | --- | --- |
|  | Cp/rxn^a^ | Cp/rxn^a^ | Bias (%)^b^ | Cp/rxn^a^ | Bias (%)^b^ | Cp/rxn^a^ | Bias (%)^b^ | Cp/rxn^a^ | Bias (%)^b^ |  |
| DBN-09004-6 | 10528 | 9921 | -5.8 | 10964 | 4.1 | 11712 | 11.2 | 10808 | 2.7 | 18.1 |
| GMB151 | 14013 | 12273 | -12.4 | 14222 | 1.5 | 14434 | 3.0 | 14709 | 5.0 | 13.0 |
| SYHT0H2 | 11630 | 15878 | 36.5 | 12491 | 7.4 | 14271 | 22.7 | 12485 | 7.4 | 16.1 |
| MON87751 | 13243 | 13895 | 4.9 | 11538 | -12.9 | 15270 | 15.3 | 13348 | 0.8 | 13.4 |
| Le1 | 530297 | 554492 | 4.6 | 534272 | 0.8 | 561592 | 5.9 | 548956 | 3.5 | 4.1 |
| 305423 | 10321 | 10982 | 6.4 | 10198 | -1.2 | 11671 | 13.1 | 10658 | 3.3 | 15.9 |
| BPS-CV127-9 | 13206 | 13405 | 1.5 | 12068 | -8.6 | 14004 | 6.0 | 14479 | 9.6 | 12.8 |
| MON87769 | 11803 | 11697 | -0.9 | 11618 | -1.6 | 10649 | -9.8 | 11556 | -2.1 | 15.5 |
| MON87701 | 12273 | 11918 | -2.9 | 13154 | 7.2 | 14099 | 14.9 | 14205 | 15.7 | 14.9 |
| MON87708 | 14158 | 13935 | -1.6 | 11114 | -21.5 | 12740 | -10.0 | 10914 | -22.9 | 16.2 |
| 40-3-2 | 12927 | 13755 | 6.4 | 14818 | 14.6 | 15036 | 16.3 | 12431 | -3.8 | 14.3 |
| A5547-127 | 13388 | 13970 | 4.4 | 14094 | 5.3 | 13201 | -1.4 | 14393 | 7.5 | 14.8 |
| A2704-12 | 12955 | 13796 | 6.5 | 11943 | -7.8 | 12169 | -6.1 | 14765 | 14.0 | 14.6 |
| MON89788 | 10957 | 13114 | 19.7 | 14031 | 28.1 | 13148 | 20.0 | 11626 | 6.1 | 20.4 |
| MON87705 | 11482 | 12174 | 6.0 | 10643 | -7.3 | 11685 | 1.8 | 11653 | 1.5 | 10.0 |
| FG72 | 14904 | 14087 | -5.5 | 14123 | -5.2 | 13926 | -6.6 | 14931 | 0.2 | 14.8 |
| DAS-68416-4 | 13070 | 10970 | -16.1 | 11673 | -10.7 | 12600 | -3.6 | 12320 | -5.7 | 14.5 |
| 356043 | 10574 | 10994 | 4.0 | 8514 | -19.5 | 9992 | -5.5 | 12609 | 19.3 | 17.7 |
| DAS-81419-2 | 11450 | 11227 | -2.0 | 10778 | -5.9 | 12565 | 9.7 | 11714 | 2.3 | 15.8 |
| DAS-44406-6 | 9969 | 9167 | -8.0 | 11200 | 12.4 | 11571 | 16.1 | 10295 | 3.3 | 17.3 |

^a^The experimental results are presented as the mean target copy numbers per analyzed reaction volume (cp/rxn), normalized to undiluted sample conditions, based on 4 total technical replicates across 2 sample dilutions.

^b^The bias for each condition is calculated in comparison to the normal conditions. Underlined number – bias >30%.

^c^The CV is calculated from all the replicates of all conditions for a given target.


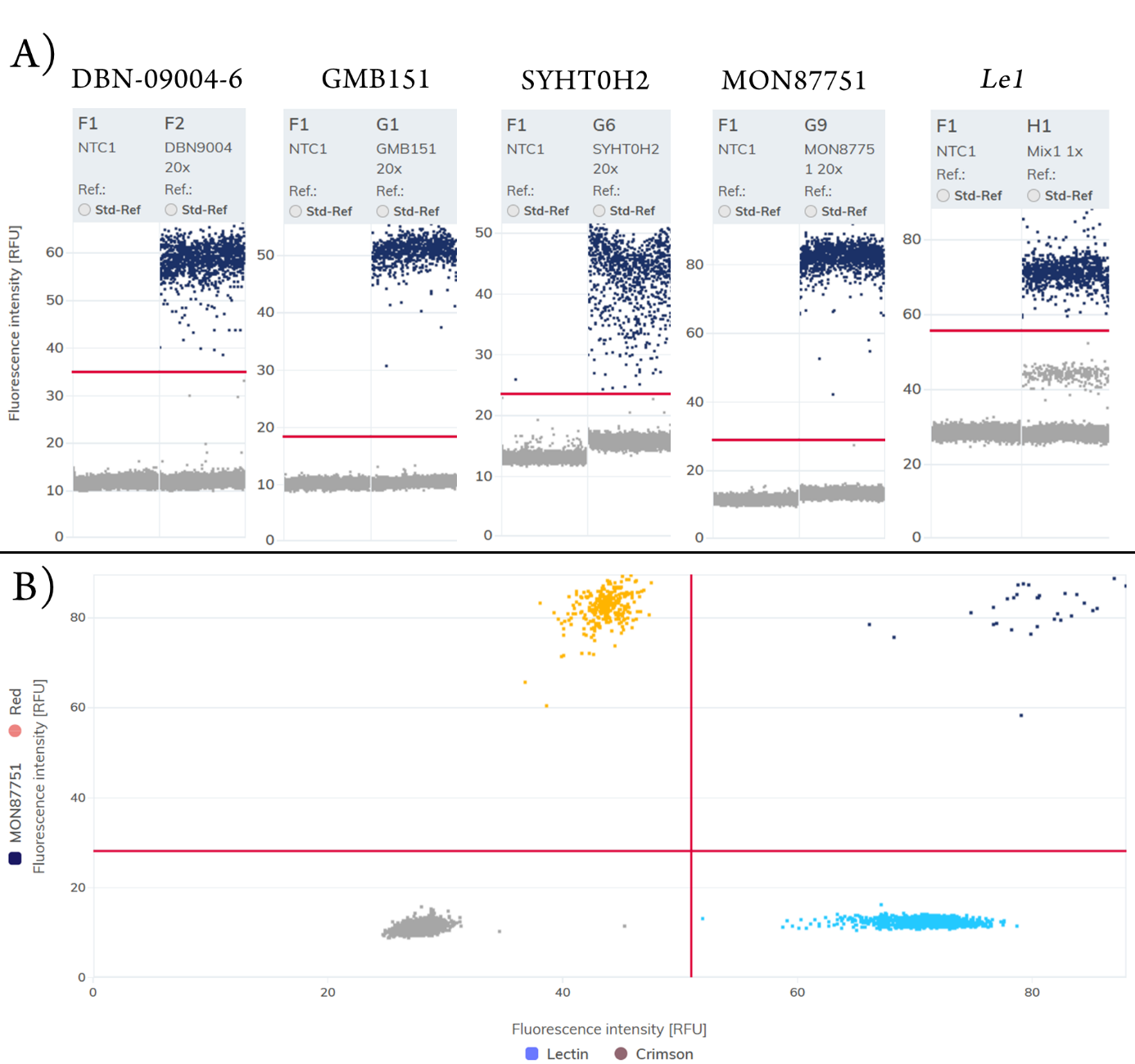


**Figure S1.** Examples of positive and negative experimental results for the 5-plex 1 assay. A) 1D amplification plots for each of the targets. The well F1 contained the no template control (NTC)–negative result, and the wells F2, G1, G6, and G9 contained DNA of soybean GM events DBN-09004-6, GMB151, SYHT0H2, and MON87751, respectively–positive results. The well H1 contained a mixture of these events. Individual partitions are plotted along the x-axis, and the y-axis represents the fluorescence intensity in relative fluorescence units (RFU). Fluorescence thresholds (red line) are set to separate the negative (gray) and positive (dark blue) partition populations. B) 2D amplification plot for the well G9, plotting partitions in terms of fluorescence detected in the crimson (target *Le1*; x-axis) and red (target MON87751; y-axis) detection channels. The partitions are clustered into four distinct populations: double negative (gray), *Le1* single positive (light blue), MON87751 single positive (yellow), and double positive (dark blue). Note the MON87751 single positive population leaning into the *Le1* positive portion of the plot. This is due to crosstalk between the red and crimson channels (seen as an additional negative population in the crimson channel 1D plot in panel A). We offset this by appropriate threshold setting. Newer versions of the QIAcuity software suite allow performing color compensation to offset crosstalk between channels.


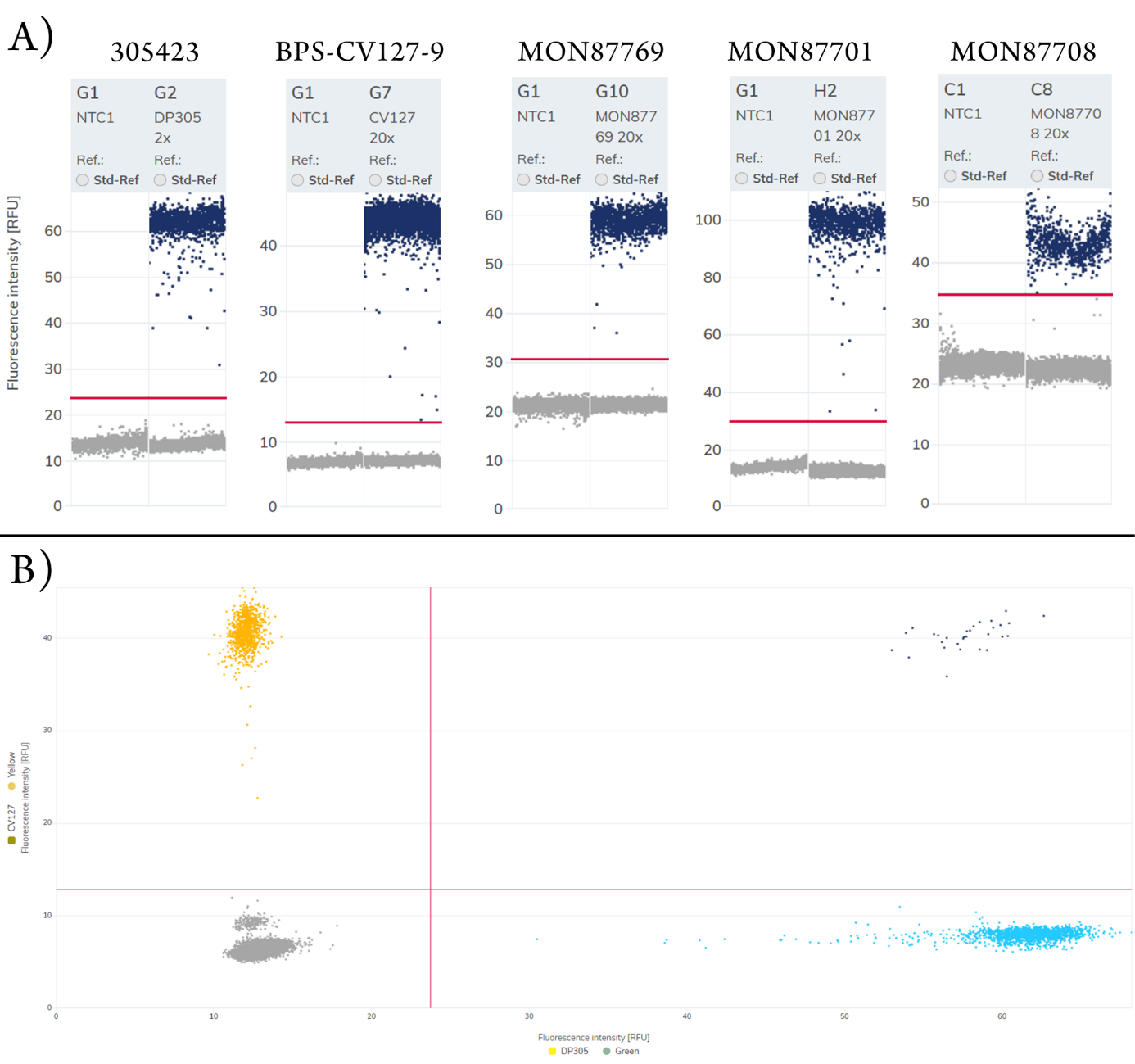


**Figure S2**. Examples of positive and negative experimental results for the 5-plex 2 assay. A) 1D amplification plots for each of the targets. The wells G1 and C1 contained the no template control (NTC)–negative result, and the wells G2, G7, G10, H2 and C8 contained DNA of soybean GM events 305423, BPS-CV127-9, MON87769, MON87701, and MON87708, respectively–positive results. Fluorescence thresholds (red line) are set to separate the negative (gray) and positive (dark blue) partition populations. B) 2D amplification plot for a sample containing a DNA mixture of aforementioned GM events, plotting partitions in terms of fluorescence detected in the green (target 305423; x-axis) and yellow (target CV127; y-axis) detection channels. The partitions are clustered into four distinct populations: double negative (gray), 305423 single positive (light blue), CV127 single positive (yellow), and double positive (dark blue).


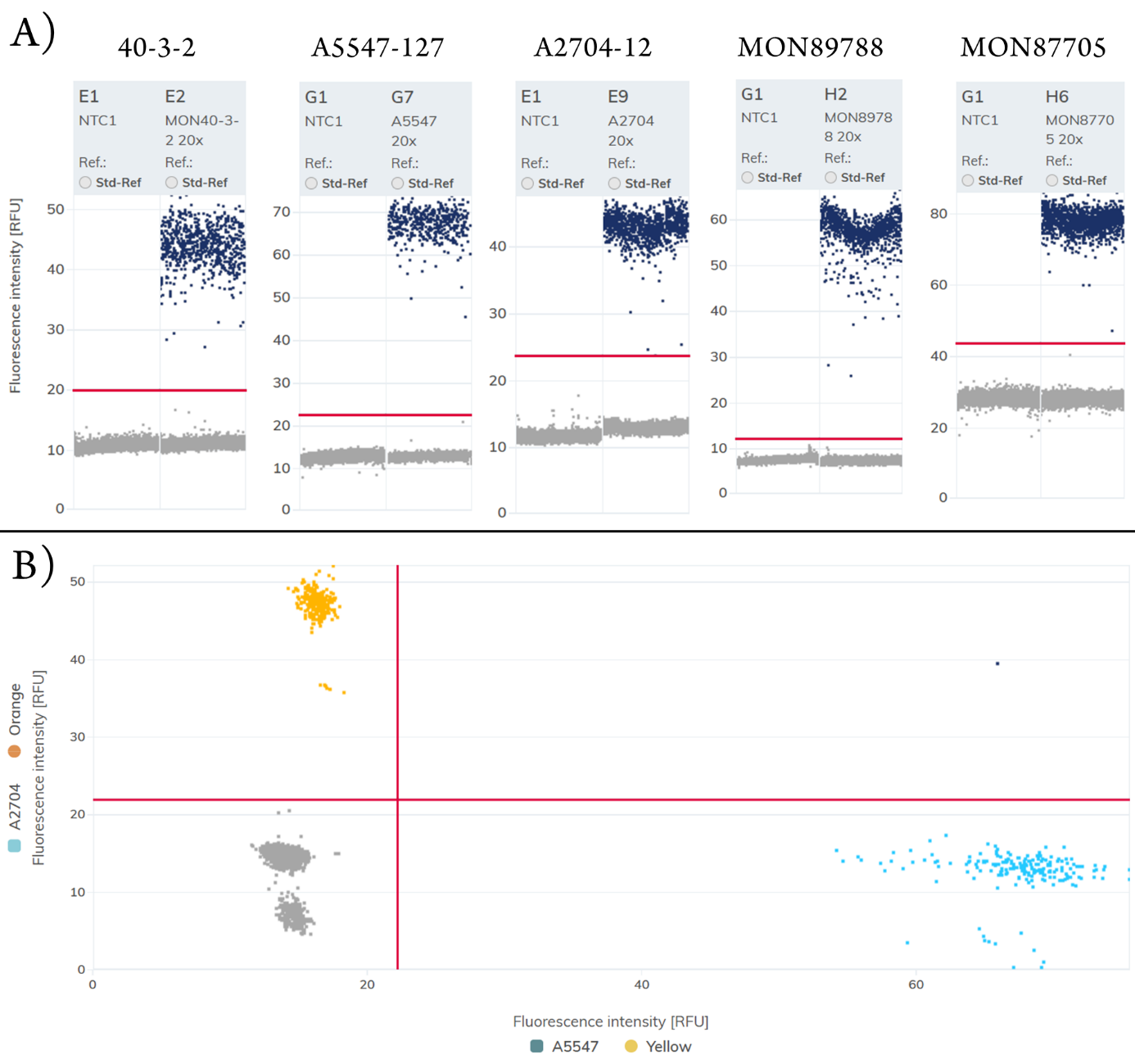


**Figure S3**. Examples of positive and negative experimental results for the 5-plex 3 assay. A) 1D amplification plots for each of the targets. The wells E1 and G1 contained the no template control (NTC) – negative result, and the wells E2, G7, E9, H2 and H6 contained DNA of soybean GM events 40-3-2. A5547-127, A2704-12, MON89788, and MON87705, respectively–positive results. Fluorescence thresholds (red line) are set to separate the negative (gray) and positive (dark blue) partition populations. B) 2D amplification plot for a sample containing a mixture of aforementioned GM events, plotting partitions in terms of fluorescence detected in the yellow (target A5547-127; x-axis) and orange (target A2704-12; y-axis) detection channels. The partitions are clustered into four distinct populations: double negative (gray), A5547-127 single positive (light blue), A2704-12 single positive (yellow), and double positive (dark blue).


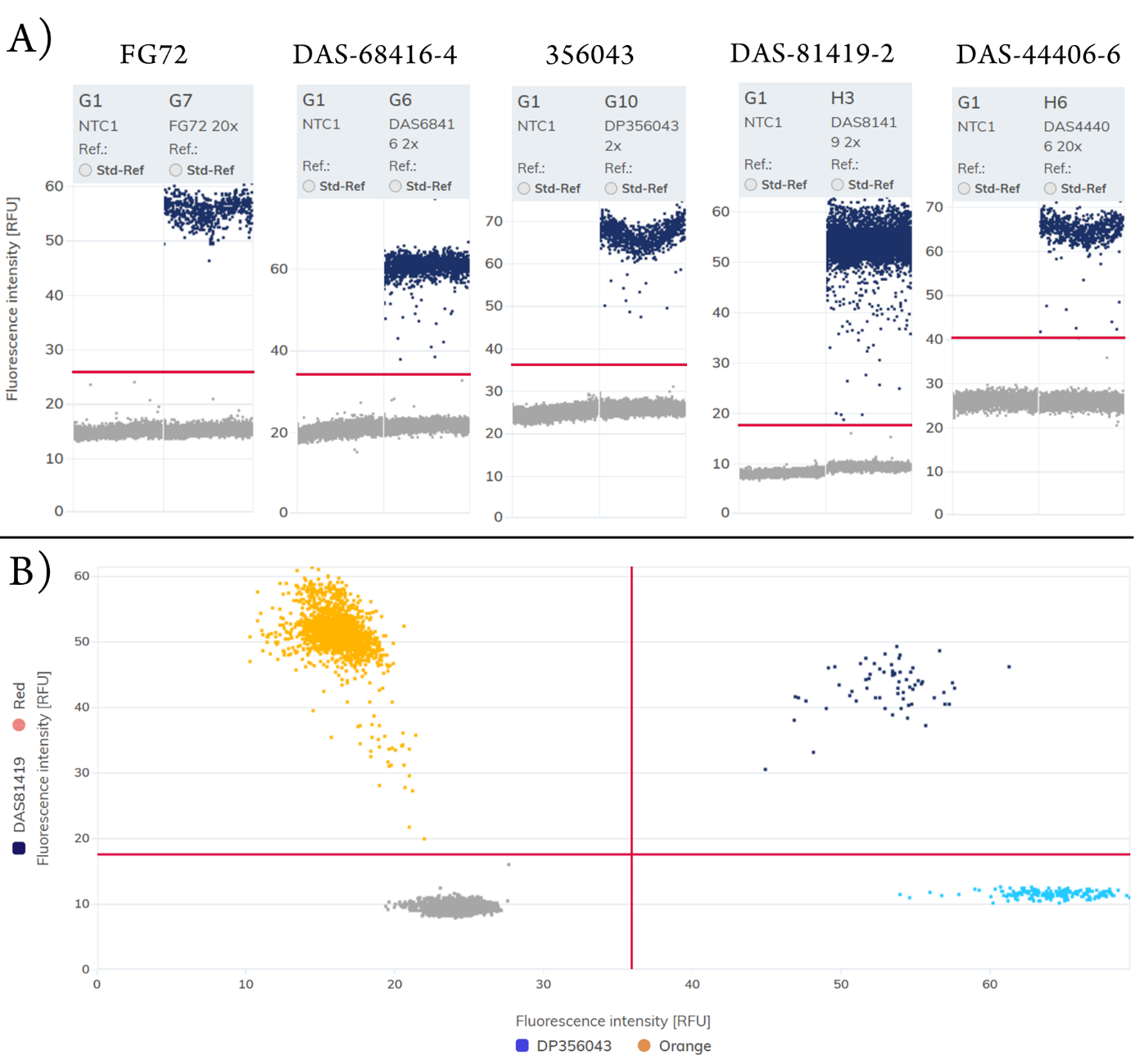


**Figure S4.** Examples of positive and negative experimental results for the 5-plex 3 assay. A) 1D amplification plots for each of the targets. The well G1 contained the no template control (NTC)–negative result, and the wells G7, G6, G10, H3, and H6 contained DNA of soybean GM events FG72. DAS-68416-4, 356043, DAS-81419-2, and DAS-44406-6, respectively–positive results. Fluorescence thresholds (red line) are set to separate the negative (gray) and positive (dark blue) partition populations. B) 2D amplification plot for a sample containing a mixture of aforementioned GM events, plotting partitions in terms of fluorescence detected in the orange (target 356043; x-axis) and red (target DAS-81419-2; y-axis) detection channels. The partitions are clustered into four distinct populations: double negative (gray), 356043 single positive (light blue), DAS-81419-2 single positive (yellow), and double positive (dark blue).


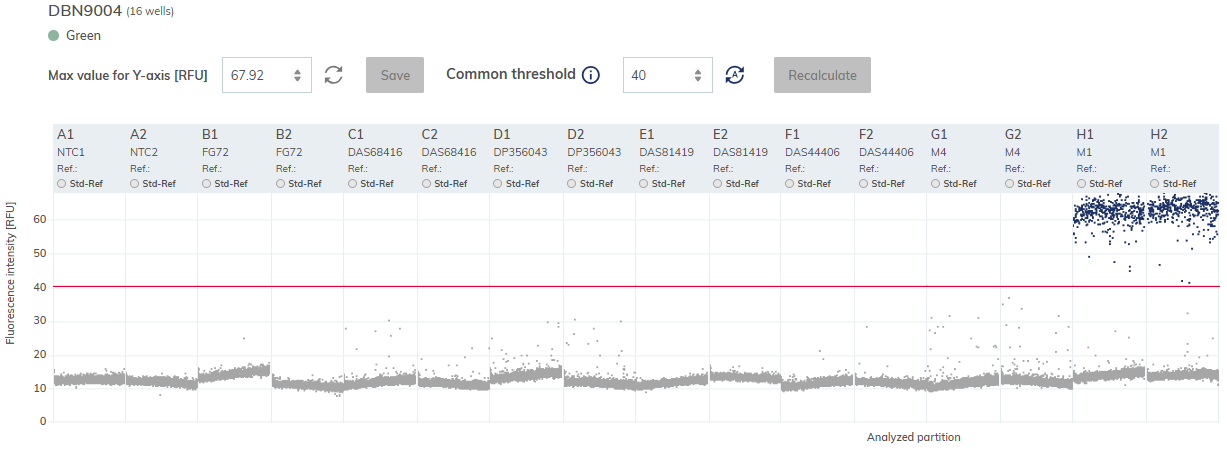


Figure S5. Low-level fluorescence in some partitions for DBN-09004-6 target when using 5-plex 1 assay with single GM line samples and complex mixtures of soybean GM lines. Wells A1 and A2 contained the non-template controls (NTC), wells B1 and B2 contained GM soybean event FG72 DNA, wells C1 and C2 contained event DAS-68416-4 DNA, wells D1 and D2 contained event 356043 DNA, wells E1 and E2 contained event DAS-81419-2 DNA, wells F1 and F2 contained event DAS-44406-4 DNA, wells G1 and G2 contained a DNA mixture of GM soybean events FG72, DAS-68416-4, 356043, DAS-81419-2 and DAS-44406-4, and wells H1 and H2 contained a DNA mixture of GM soybean events detected by 5-plex 1 (DBN-09004-6, GMB151, SYHT0H2 and MON87701). Individual partitions are plotted along the x-axis, and the y-axis represents the fluorescence intensity in relative fluorescence units (RFU). The threshold (red line) is set in a way to separate true positives from negative signal.


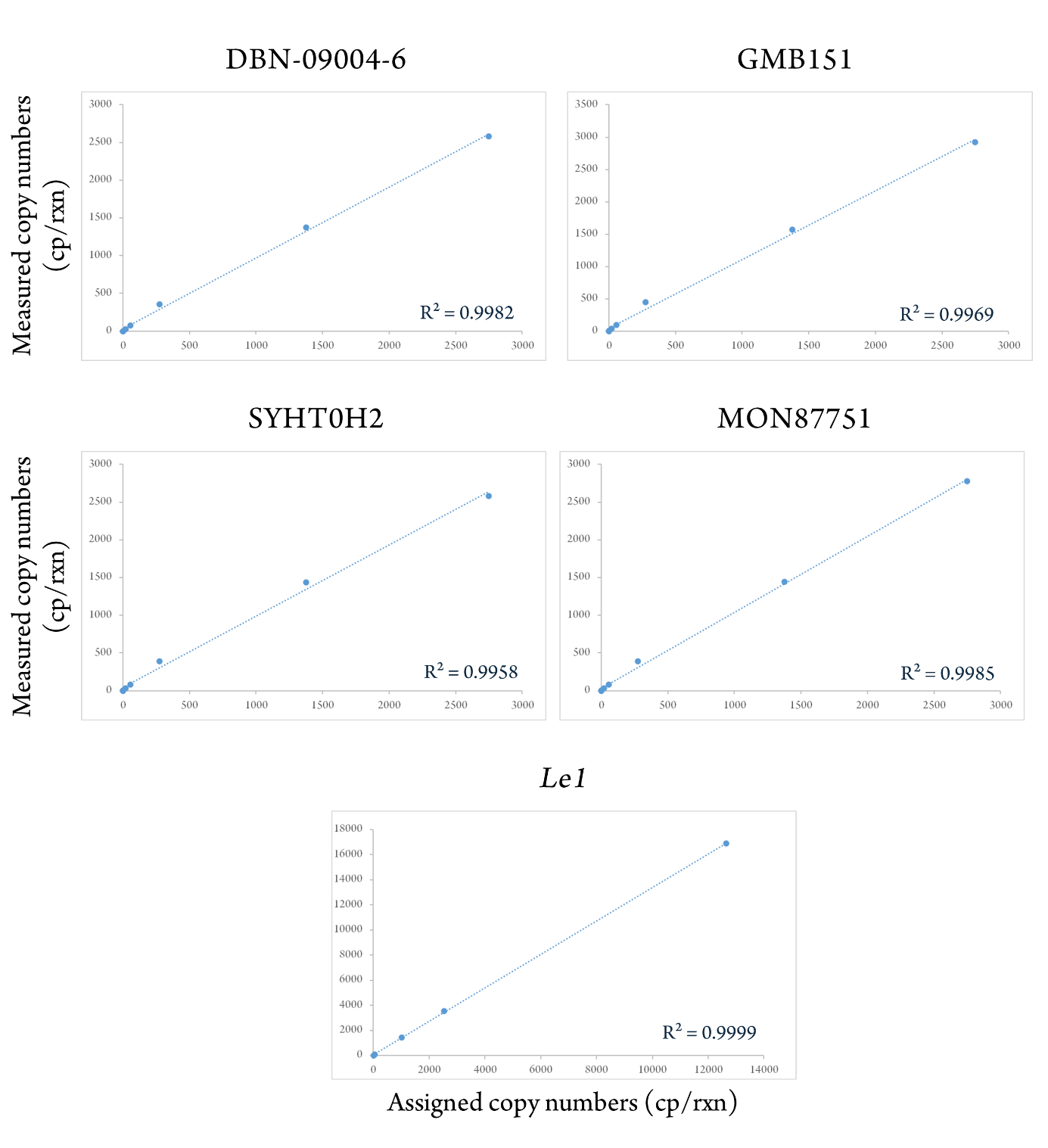


**Figure S6.** Linearity of the 5-plex 1 assay for each target. Measured copy numbers are compared to assigned copy numbers for serial dilutions of GM soybean mixture. cp/rxn – copies per reaction.


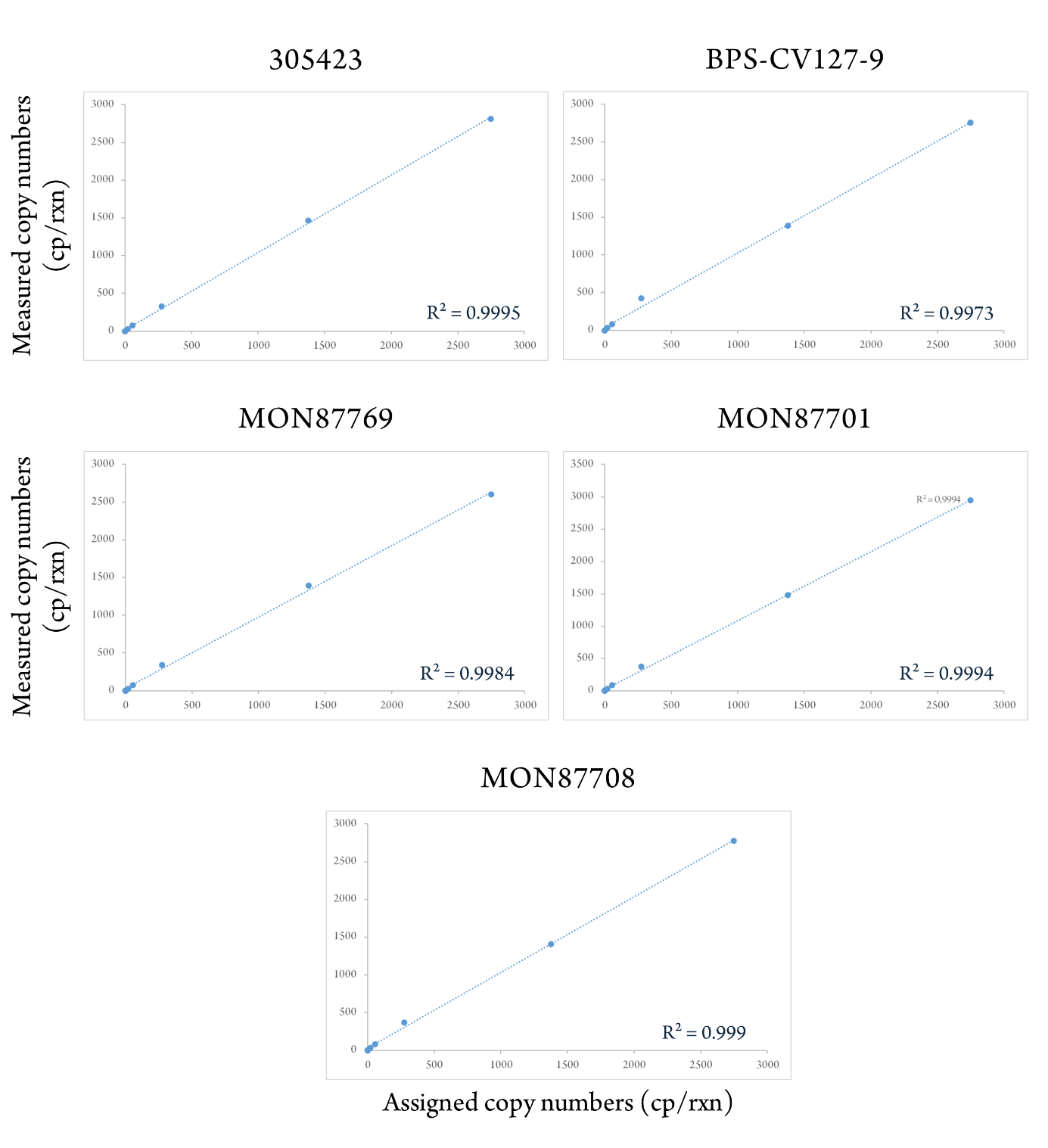


**Figure S7.** Linearity of the 5-plex 2 assay for each target. Measured copy numbers are compared to assigned copy numbers for serial dilutions of GM soybean mixture. cp/rxn – copies per reaction.


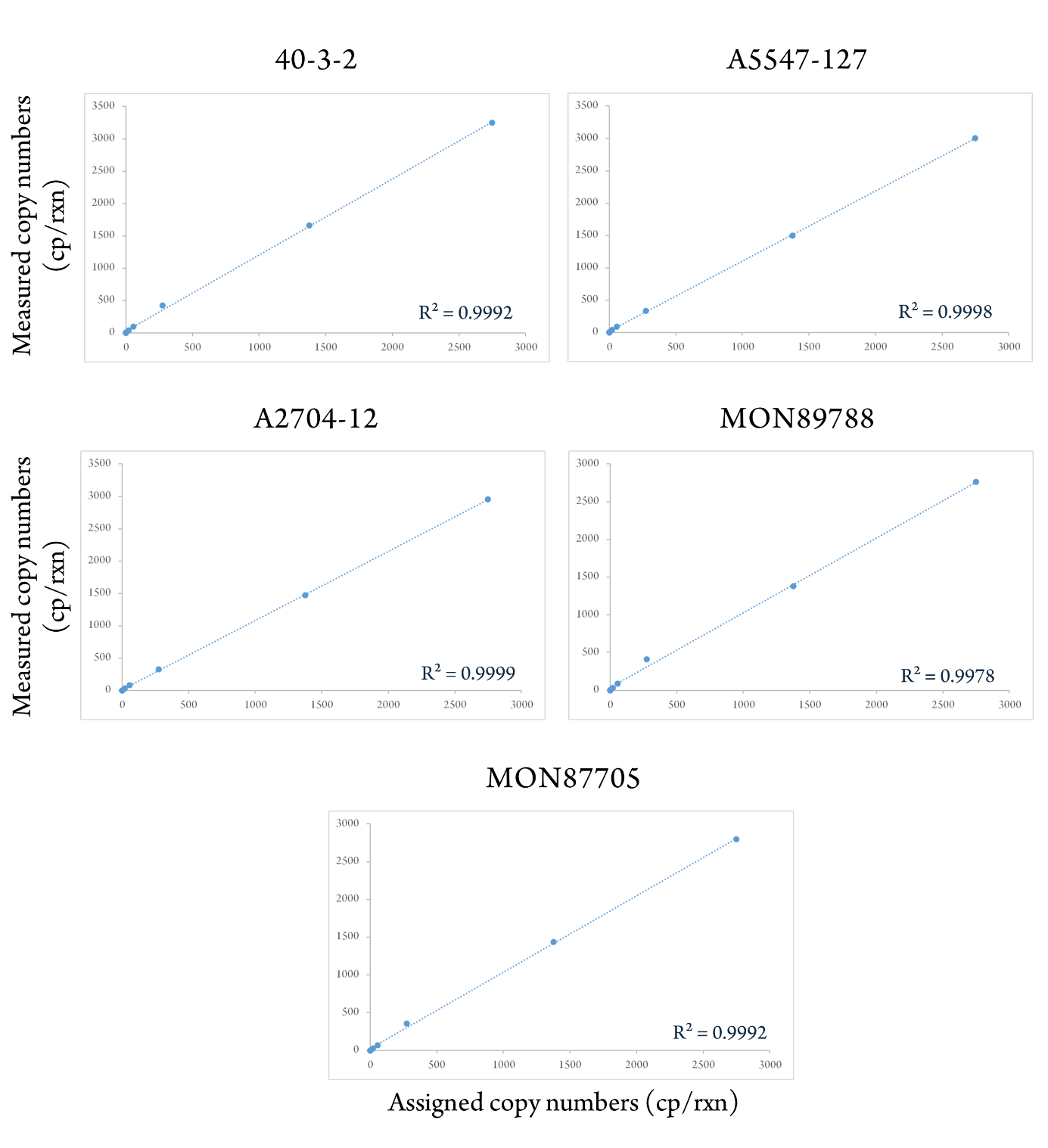


**Figure S8.** Linearity of the 5-plex 3 assay for each target. Measured copy numbers are compared to assigned copy numbers for serial dilutions of GM soybean mixture. cp/rxn – copies per reaction.


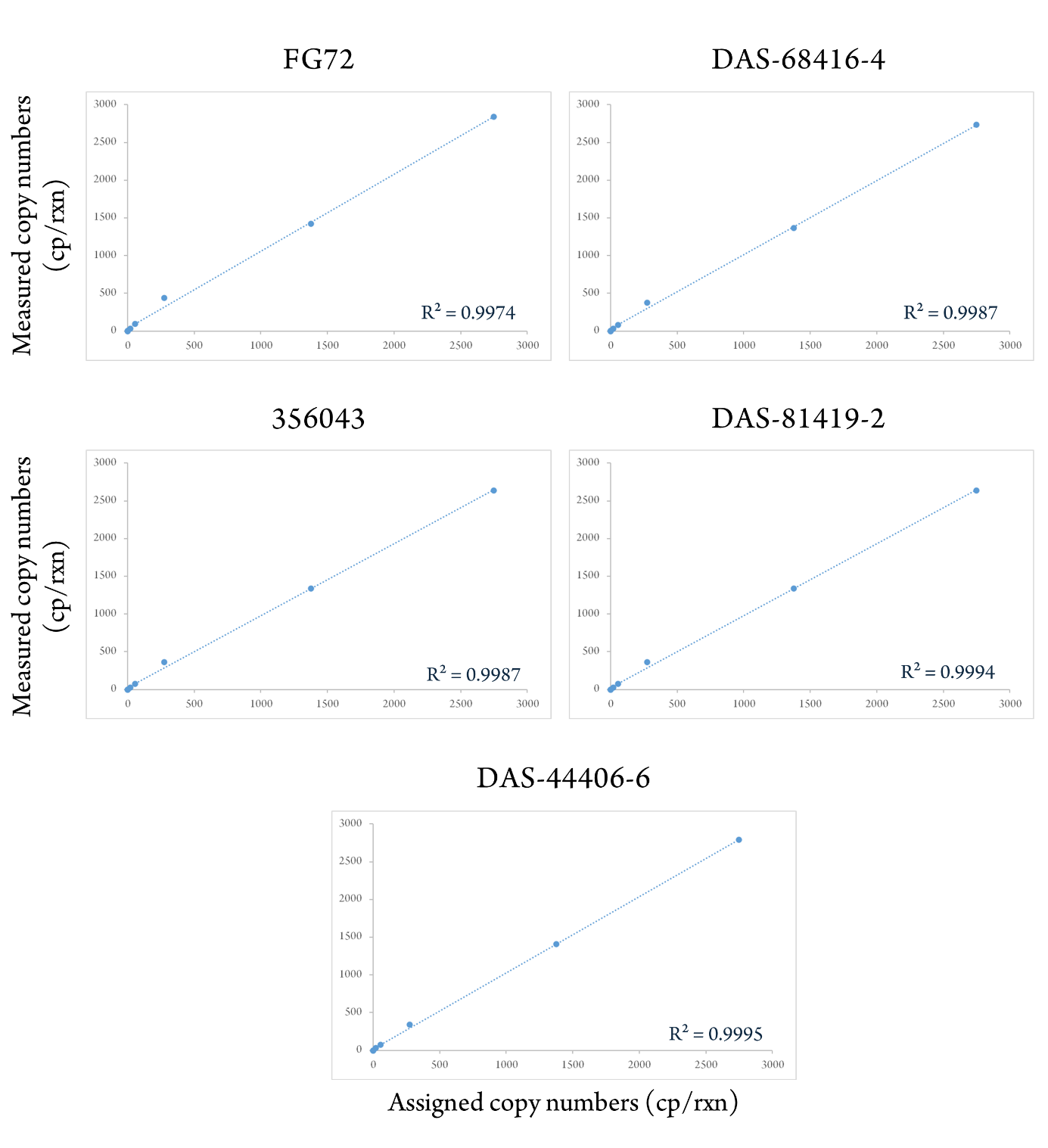


**Figure S9.** Linearity of the 5-plex 4 assay for each target. Measured copy numbers are compared to assigned copy numbers for serial dilutions of GM soybean mixture. cp/rxn – copies per reaction.
