# Supplementary material for: Simple, fast, reliable: multiplex digital PCR quantification of 19 genetically modified soybean events
Source: GM Crops Food. 2026 Feb 27;17(1):2635816. doi: 10.1080/21645698.2026.2635816 (PMC12959185; doi:10.1080/21645698.2026.2635816)
Supplement: Supplementary information 2_dMIQE2020 checklist.docx [file KGMC_A_2635816_SM9267.docx]

SuppLEMENTARY infromation

Simple, Fast, Reliable: Multiplex Digital PCR Quantification of 19 Genetically Modified Soybean Events

Amadej Jelenčič^a,b^, Dejan Štebih^a^, Tina Demšar^a^ and David Dobnik^a^

^a^Department of Biotechnology and Systems Biology, National Institute of Biology, Večna pot 121, SI-1000 Ljubljana, Slovenia

^b^Jozef Stefan International Postgraduate School, Jamova 39, SI-1000 Ljubljana, Slovenija

KEYWORDS: genetically modified (GM) crops, transgenic, soybean, Glycine max, quantification, digital PCR (dPCR), multiplex, multi-target

Minimum Information for Publication of Quantitative Digital PCR Experiments for 2020 (dMIQE2020) checklist

| ITEM TO CHECK | PROVIDED | COMMENT |
| --- | --- | --- |
|  | Y/N |  |
| 1. SPECIMEN |  |  |
| Detailed description of specimen type and numbers | Y | Materials and methods, section 2.1 Test materials |
| Sampling procedure (including time to storage) | N |  |
| Sample aliquotation, storage conditions and duration | Y | Materials and methods, section 2.2 DNA extraction |
| 2. NUCLEIC ACID EXTRACTION |  |  |
| Description of extraction method including amount of sample processed | Y | Materials and methods, section 2.2 DNA extraction |
| Volume of solvent used to elute/resuspend extract | Y | Materials and methods, section 2.2 DNA extraction |
| Number of extraction replicates | Y | Materials and methods, section 2.2 DNA extraction |
| Extraction blanks included? | Y | Materials and methods, section 2.2 DNA extraction |
| 3. NUCLEIC ACID ASSESSMENT AND STORAGE |  |  |
| Method to evaluate quality of nucleic acids | N |  |
| Method to evaluate quantity of nucleic acids (including molecular weight and calculations when using mass) | N |  |
| Storage conditions: temperature, concentration, duration, buffer, aliquots | Y | Materials and methods, section 2.2 DNA extraction |
| Clear description of dilution steps used to prepare working DNA solution | Y | Materials and methods |
| 4. NUCLEIC ACID MODIFICATION | NA | No modifications were performed |
| Template modification (digestion, sonication, pre-amplification, bisulphite etc.) |  |  |
| Details of repurification following modification if performed |  |  |
| 5. REVERSE TRANSCRIPTION | NA |  |
| cDNA priming method and concentration |  |  |
| One or two step protocol (include reaction details for two step) |  |  |
| Amount of RNA added per reaction |  |  |
| Detailed reaction components and conditions |  |  |
| Estimated copies measured with and without addition of RT* |  |  |
| Manufacturer of reagents used with catalogue and lot numbers |  |  |
| Storage of cDNA: temperature, concentration, duration, buffer and aliquots |  |  |
| 6. dPCR OLIGONUCLEOTIDES DESIGN AND TARGET INFORMATION |  |  |
| Sequence accession number or official gene symbol | N | NA |
| Method (software) used for design and *in silico* verification | Y | Materials and methods, section 2.3 *In silico* Specificity and Primer/Probe Interaction Prediction |
| Location of amplicon | N | NA |
| Amplicon length | Y | Supplementary table 1 |
| Primer and probe sequences (or amplicon context sequence)** | Y | Supplementary table 1 |
| Location and identity of any modifications | N | Supplementary table 1 |
| Manufacturer of oligonucleotides | Y | ... |
| 7. dPCR PROTOCOL |  |  |
| Manufacturer of dPCR instrument and instrument model | Y | Materials and methods, section 2.4 Digital PCR on the QIAcuity Platform |
| Buffer/kit manufacturer with catalogue and lot number |  | Materials and methods, section 2.4 Digital PCR on the QIAcuity Platform |
| Primer and probe concentration |  | Materials and methods, section 2.4 Digital PCR on the QIAcuity Platform |
| Pre-reaction volume and composition (incl. amount of template and if restriction enzyme added) | Y | Materials and methods, section 2.4 Digital PCR on the QIAcuity Platform |
| Template treatment (initial heating or chemical denaturation) | N | No template treatment prior to dPCR |
| Polymerase identity and concentration, Mg++ and dNTP concentrations*** | N | QuantiNova DNA polymerase (QIAGEN); concentrations not disclosed by manufacturer (QIAcuity Probe PCR Kit, Cat. No. / ID:  250102) |
| Complete thermocycling parameters | Y | Materials and methods, section 2.4 Digital PCR on the QIAcuity Platform |
| 8. ASSAY VALIDATION |  |  |
| Details of optimisation performed | N | All assays were validated by EURL thus a validation of simplex assays was not repeated in this study. |
| Analytical specificity (vs. related sequences) and limit of blank (LOB) | N | All assays were validated by EURL thus a validation of simplex assays was not repeated in this study. |
| Analytical sensitivity/LoD and how this was evaluated | N | All assays were validated by EURL thus a validation of simplex assays was not repeated in this study. |
| Testing for inhibitors (from biological matrix/extraction) | N | All assays were validated by EURL thus a validation of simplex assays was not repeated in this study. |
| 9. DATA ANALYSIS |  |  |
| Description of dPCR experimental design | Y | Materials and methods |
| Comprehensive details negative and positive of controls (whether applied for QC or for estimation of error) | Y | Materials and methods |
| Partition classification method (thresholding) | Y | Materials and methods |
| Examples of positive and negative experimental results (including fluorescence plots in supplemental material) | Y | Supporting information 1 (Figures S1-S4) |
| Description of technical replication | Y | Materials and methods |
| Repeatability (intra-experiment variation) | Y | Materials and methods |
| Reproducibility (inter-experiment/user/lab etc. variation ) | Y | Materials and methods |
| Number of partitions measured (average and standard deviation ) | raw data available on request |  |
| Partition volume | raw data available on request |  |
| Copies per partition (λ or equivalent ) (average and standard deviation) | raw data available on request |  |
| dPCR analysis program (source, version) | Y | Materials and methods, section 2.4 Digital PCR on the QIAcuity Platform |
| Description of normalisation method | Y | Materials and methods |
| Statistical methods used for analysis | Y | Materials and methods |
| Data transparency | raw data available on request |  |
